# Culturable Fungi Recovered from Deep-Sea Sediments: Diversity, Phylogenetic Placement, and In Vitro Antagonistic Activity Against Phytopathogenic Fungi

**DOI:** 10.3390/jof12080544

**Published:** 2026-07-23

**Authors:** Xiaoxiong Xu, Zhongmin Lin, Tianyou Liao, Jiacheng Guo, Jiajun Li, Jianping Li, Hanqing Wang, Huimin Feng, Kexian Yi

**Affiliations:** 1 School of Life and Health Sciences, Hainan International One Health Institute, Hainan University, Haikou 570228, China; xuxiaoxiong@hntou.edu.cn; 2 Key Laboratory for Tropical Crop Molecular Breeding, Hainan Tropical Ocean University, Sanya 572022, China; 18789386706@163.com (Z.L.); lty2975580654@163.com (T.L.); kfdmgbz0015@163.com (J.G.); lijiajun19970226@163.com (J.L.); a3491280941@126.com (J.L.); 18715691901@163.com (H.W.); 3 Sanya Research Institute, Chinese Academy of Tropical Agricultural Sciences, Sanya 572025, China; 4 Environment and Plant Protection Institute, Chinese Academy of Tropical Agricultural Sciences, Haikou 571101, China

**Keywords:** deep-sea sediments, culturable fungi, ITS phylogeny, fungal diversity, low-similarity phylotypes, antifungal activity, volatile organic compounds

## Abstract

Deep-sea sediment-derived fungal isolates remain underrepresented in organized living collections for phenotype-based screening. We applied 12 complementary isolation protocols—dilution plating, ethylenediaminetetraacetic acid (EDTA) pretreatment, and plate stamping—to four deep-sea sediment materials (2595–6000 m) representing three sampling units from the Mariana Trench slope region and the South China Sea. After morphotype-based purification, isolates were characterized by internal transcribed spacer (ITS) closest-match analysis and phylogenetic placement; selected representatives were further screened against five phytopathogenic strains using dual-culture, culture filtrate, and volatile organic compound (VOC) assays. In total, 159 isolates were placed within Ascomycota, Basidiomycota, and Mucoromycota, representing 35 closest-match genus-level groups and 22 low-similarity ITS phylotypes (<95% similarity) preserved as living cultures. *Cladosporium*, *Penicillium*, and *Aspergillus* were most frequent. Recovered diversity profiles varied among sediment materials and protocols, with plate stamping capturing a large, compositionally distinct subset of the collection. In dual culture, all 23 tested isolates inhibited at least one pathogen, with maximum mycelial growth inhibition reaching 98.74%. Culture filtrate and VOC assays produced partially discordant activity profiles, highlighting assay-format-dependent effects. This study establishes an ITS-organized living fungal collection and provides a phenotype-based framework for targeted taxonomic, chemical, and biocontrol-oriented follow-up.

## 1. Introduction

Deep-sea sediments constitute one of the most extensive microbial habitats on Earth and have repeatedly yielded fungal signatures in molecular surveys. Sequence-based studies have revealed diverse fungal assemblages in sediments from the Central Indian Basin, the Pacific Ocean, and other deep-sea regions, including lineages with limited taxonomic affinity to described taxa [1,2,3]. These culture-independent approaches are indispensable for resolving community structure and detecting uncultured diversity; however, they do not provide living material for morphological characterization, multilocus taxonomic refinement, physiological study, or functional screening. Culture-dependent and culture-independent approaches are therefore complementary in fungal discovery and systematics [4,5].

Despite this growing body of evidence, the culturable fungal fraction of deep-sea sediments remains less documented than its bacterial and archaeal counterparts. Previous culture-dependent studies have recovered diverse fungi from deep-sea sediments of the Central Indian Basin, the South China Sea, the South Atlantic Ocean, and other regions, with Ascomycota often dominating the recovered assemblages and genera such as *Aspergillus*, *Penicillium*, and *Cladosporium* frequently reported [5,6,7]. Nevertheless, the composition of culturable fungal collections can be strongly influenced by sampling locality, sediment properties, and methodological choices, including medium composition, dilution regime, pretreatment, incubation conditions, and colony selection strategy. Because cultivation conditions influence which propagules germinate and are retained as pure cultures, comparing multiple protocols on the same sediment materials can help describe method-dependent recovery patterns in the culturable fraction. These considerations provide the rationale for examining culturable fungi from both the Mariana Trench slope region and the South China Sea using complementary isolation protocols.

Molecular identification underpins the interpretation of culturable fungal collections, but the level of taxonomic resolution depends on the marker and fungal group examined. The internal transcribed spacer (ITS) region is widely accepted as the primary fungal DNA barcode and is useful for first-pass identification and phylogenetic placement of environmental isolates [8]. However, ITS alone is often insufficient for species-level resolution in species-rich and taxonomically complex genera, including *Aspergillus*, *Penicillium*, *Cladosporium*, and *Talaromyces*, for which multilocus datasets and morphological characters are commonly required [9,10,11,12]. Accordingly, low ITS similarity to reference sequences should be treated as an indication of divergent or poorly resolved phylotypes that warrant further taxonomic investigation, rather than as direct evidence for novel species. Within this context, ITS-based phylogenetic placement remains a practical and informative first step for organizing culturable collections and prioritizing isolates for future taxonomic refinement.

Living fungal collections also provide opportunities for phenotype-based functional screening and for prioritizing strains with biocontrol-related traits. Fungi recovered from deep-sea and marine-associated samples have attracted attention as sources of bioactive secondary metabolites, including compounds with antimicrobial, cytotoxic, antioxidant, and other biological activities [13,14]. However, much of this work has emphasized pharmaceutical or general antimicrobial activity, whereas less attention has been given to screening culturable fungi recovered from deep-sea sediments against plant-pathogenic fungi. Plant-pathogenic fungi remain major constraints on crop production, and in vitro assays can provide an initial assessment of antagonistic traits in candidate fungal isolates [15]. Dual-culture assays, culture filtrate bioassays, and volatile organic compound (VOC) assays probe different interaction contexts, including direct confrontation, filtrate-associated non-contact effects, and volatile-mediated effects. Such assays do not establish field-level biocontrol efficacy, but they are useful for prioritizing strains for downstream mechanistic studies and further evaluation of biocontrol-related traits. Fungal volatile organic compounds, in particular, have received increasing attention as antifungal agents, although their effects are often strain-, pathogen-, and condition-dependent [16].

In this study, we applied 12 complementary isolation protocols—dilution plating, ethylenediaminetetraacetic acid (EDTA)-pretreated dilution plating, and plate stamping—to four deep-sea sediment materials collected at depths of 2595–6000 m from the Mariana Trench slope region and the South China Sea. These materials comprised three sampling units, with DS03 and DS04 derived from different sediment fractions at the same South China Sea station and retained as operationally separate isolation sources. Using ITS closest-match analysis and phylogenetic placement, we established an ITS-organized living collection and identified low-similarity ITS phylotypes preserved as cultures for future taxonomic refinement. Representative isolates were further screened against five phytopathogenic strains—*Colletotrichum gloeosporioides*, *Ganoderma pseudoferreum*, and *Phellinus noxius*—using dual-culture, culture filtrate, and volatile organic compound assays to assess assay-format-dependent effects. By integrating protocol-resolved recovery analysis, ITS-organized resource establishment, and multi-format phenotype-based screening within the same living collection, this study provides a documented platform for targeted taxonomic, chemical, and biocontrol-oriented follow-up.

## 2. Materials and Methods

### 2.1. Sample Collection and Processing

**Figure 1:**
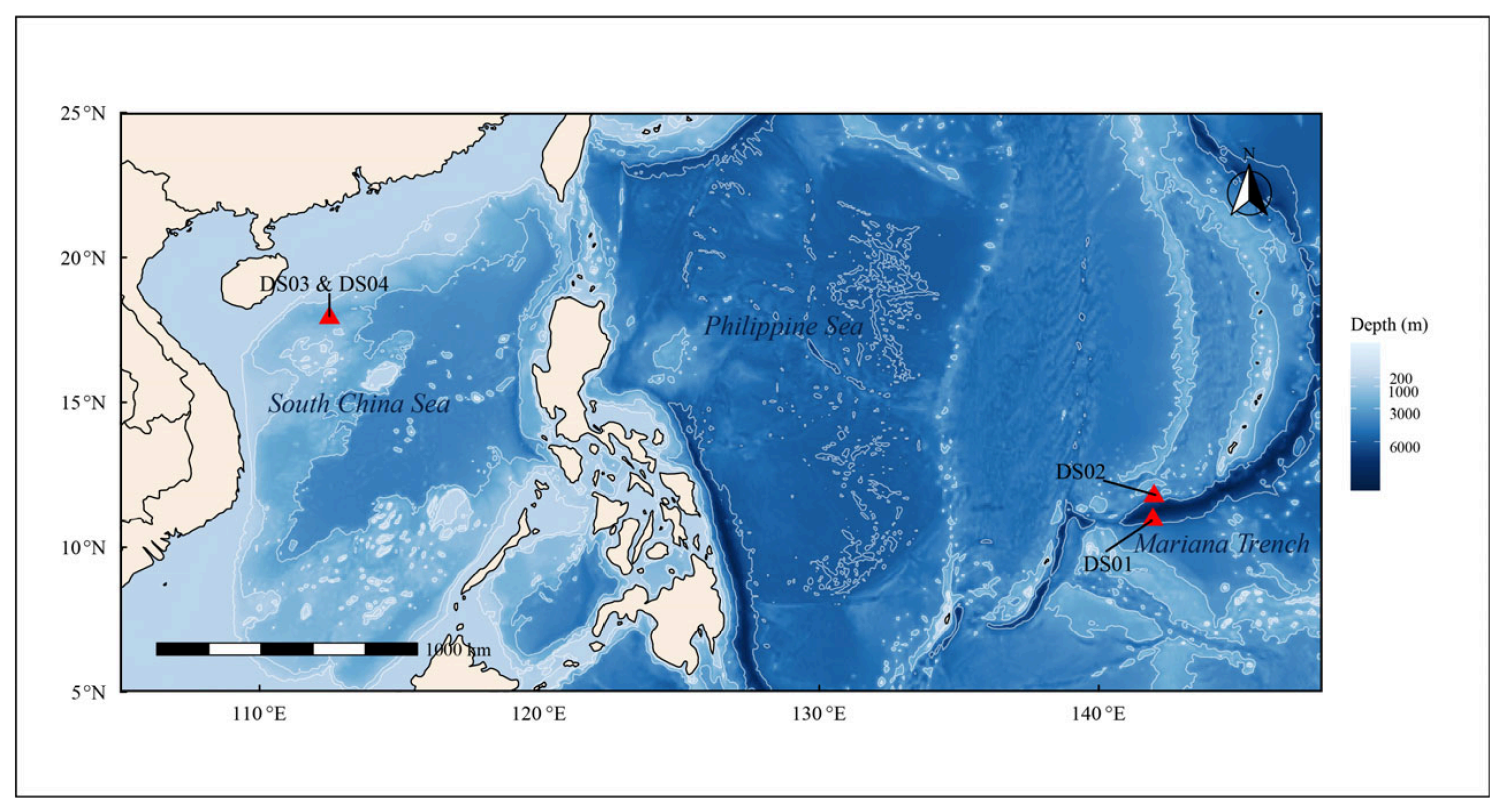
Geographic locations of the deep-sea sediment sampling sites. The map was generated in R version 4.4.1 (R Foundation for Statistical Computing, Vienna, Austria) using bathymetric data from the ETOPO 2022 Global Relief Model and coastline data from Natural Earth. Bathymetric contours at 200, 1000, 3000, and 6000 m are shown.

Four deep-sea sediment materials, designated DS01–DS04, were collected from the Challenger Deep slope region of the Mariana Trench and the deep basin of the South China Sea (Figure 1). Sampling region, water depth, geographic coordinates, and the sediment fraction used for fungal isolation are provided in Table S1. DS01 and DS02 were obtained from the Challenger Deep slope region at water depths of approximately 6000 m and 5400 m, respectively, whereas DS03 and DS04 were obtained from different sediment fractions at a single South China Sea station at approximately 2595 m water depth. All four materials were collected using sterile push corers operated in situ by submersible manipulator arms. The sediment fractions used for fungal isolation differed among materials: bulk sediment material for DS01, columnar sediment material for DS02, a mixed surface and deeper sediment portion for DS03, and a middle sediment fraction for DS04. Although DS03 and DS04 were collected from the same South China Sea station, they represented different sediment fractions and were therefore operationally treated as separate sediment sources for fungal isolation, rather than as independent sampling stations or replicate stratigraphic samples. Accordingly, comparisons among DS01–DS04 are interpreted as descriptive recovery profiles associated with operational sediment materials, not as formal tests of biogeographic, depth-related, or sediment-layer effects. After recovery, sediment materials were subsampled on board, transferred into sterile containers, and stored at −80 °C until fungal isolation. Fungal isolation was subsequently performed aseptically in a laminar-flow cabinet.

### 2.2. Isolation of Culturable Fungi

To broaden the recovery of culturable fungi, two complementary inoculation strategies—dilution plating and plate stamping—were combined with four fungal isolation media, generating 12 distinct isolation protocols (T01–T12; Table S2). These protocols were designed to sample different culturable fractions rather than to provide quantitatively equivalent comparisons of isolation efficiency, because dilution plating and plate stamping differed in inoculum preparation, sediment transfer, and propagule deposition on agar surfaces. The four media used were glucose-peptone-yeast extract agar (GPY) [17], malt extract agar (MEA) [18], Fungus No. II medium (based on the Fungus No. II medium formulation reported by Zhang et al. [19]; full composition in Table S3), and Martin’s rose bengal agar (Martin) [20]. All media were prepared with 40% (*v*/*v*) aged seawater in distilled water, adjusted to the medium-specific target pH where applicable and otherwise used at the natural pH of the formulation (Table S3), and autoclaved at 121 °C for 20 min. After cooling to approximately 50 °C, each medium was supplemented with a filter-sterilized antibiotic cocktail to suppress bacterial growth, providing final concentrations of 100 μg/mL each of chloramphenicol, streptomycin sulfate, and ampicillin. Antibiotics were included to reduce bacterial overgrowth and facilitate fungal purification; however, antibiotic supplementation may also influence the recovered culturable fraction by altering bacterial–fungal interactions or selecting fungi able to grow under antibiotic-amended conditions. Therefore, the recovered fungal assemblage should be interpreted as the fungal fraction obtained under the present antibiotic-supplemented cultivation conditions. All inoculated plates were incubated at 28 °C in the dark and inspected daily for up to 3 weeks.

#### 2.2.1. Dilution Plating

For each sediment material, 2 g of sediment was air-dried in a laminar-flow cabinet and gently ground into powder using a sterile mortar and pestle, and then suspended in 18 mL of sterile-filtered aged seawater in a 50-mL centrifuge tube to generate a 10^−1^ (*w*/*v*) suspension. To examine the effect of pretreatment on fungal recovery, two parallel 10^−1^ suspensions were prepared from each sediment material: an untreated suspension and an ethylenediaminetetraacetic acid (EDTA)-pretreated suspension. The EDTA-pretreated suspension was prepared by adding 0.8 mL of 10% (*w*/*v*) EDTA solution to the 10^−1^ sediment suspension prior to agitation [18]. Both suspensions were agitated on an orbital shaker at 150 rpm for 30 min at room temperature to disperse fungal propagules [21]. A 10^−2^ suspension was subsequently prepared by transferring 2 mL of the 10^−1^ suspension into 18 mL of sterile-filtered aged seawater.

Aliquots of 0.2 mL from each suspension were spread on agar plates. Untreated suspensions (10^−1^ and 10^−2^) were inoculated onto GPY, MEA, Fungus No. II, and Martin’s rose bengal agar (T01–T08; Table S2). EDTA-pretreated suspensions (10^−1^ and 10^−2^) were inoculated onto MEA only (T09 and T10). Each protocol was performed in three independent replicates.

#### 2.2.2. Plate Stamping

The plate-stamping protocol was adapted from Jensen et al. [22] as a complementary approach to liquid-suspension dilution methods. Briefly, 1 g of sediment was placed in a sterile Petri dish and air-dried in a laminar-flow cabinet for 1 h; aggregates were gently dispersed using a sterile mortar and pestle. An autoclaved foam plug (2 cm in diameter) was firmly pressed onto the sediment surface and then stamped sequentially onto the agar surface in a clockwise pattern, generating a decreasing inoculum gradient through progressive depletion of sediment particles and fungal propagules. Plate stamping was performed on Fungus No. II and GPY media (T11 and T12; Table S2), each in three independent replicates.

### 2.3. Morphological Grouping, Purification, and Preservation

Emerging fungal colonies from all plates were examined daily and grouped based on colony texture, color of aerial and substrate mycelia, production of soluble pigments, and growth rate [23]. Representative colonies from each distinct morphotype were picked and repeatedly subcultured on fresh potato dextrose agar (PDA) plates (composition in Table S3) until pure cultures were obtained. All purified strains were assigned unique isolate codes (e.g., DSF001) and preserved on PDA slants at 4 °C for short-term use, and as 20% (*v*/*v*) glycerol stock suspensions stored at −80 °C for long-term maintenance. Two priority isolates, DSF059 and DSF149, have been deposited in the China Center for Type Culture Collection (CCTCC, Wuhan, China) under accession numbers CCTCC No. M 20232310 and CCTCC No. M 20232230, respectively. These isolates were prioritized for formal deposition because of their taxonomic and functional significance and their use in follow-up taxonomic and application-oriented studies.

### 2.4. Molecular Identification and Phylogenetic Placement

Genomic DNA was extracted using a modified cetyltrimethylammonium bromide (CTAB) method [24] and standardized to 50 ng/μL. The ITS region was amplified by polymerase chain reaction (PCR) with primers ITS1 (5′-TCCGTAGGTGAACCTGCGG-3′) and ITS4 (5′-TCCTCCGCTTATTGATATGC-3′) [25] in 50-μL reactions containing 25 μL of 2× Taq Plus PCR Master Mix (Vazyme Biotech, Nanjing, China; P111), 2 μL of each primer (10 μM), and 2 μL of template DNA. Thermal cycling on a FlexCycler (Analytik Jena, Jena, Germany) was: 94 °C for 5 min; 30 cycles of 94 °C for 30 s, 55 °C for 30 s, and 72 °C for 1 min; 72 °C for 7 min. Products were verified by 1% agarose gel electrophoresis and sequenced bidirectionally at Majorbio Biopharm Technology (Shanghai, China). Reads were assembled and manually proofread in SeqMan in the Lasergene version 7.1 software package (DNASTAR, Madison, WI, USA).

Assembled ITS sequences were queried against the National Center for Biotechnology Information (NCBI) GenBank database using the Basic Local Alignment Search Tool for nucleotide sequences (BLASTn) web service (https://blast.ncbi.nlm.nih.gov/Blast.cgi; accessed on 22 July 2026). Provisional taxonomic assignments were made conservatively based on the closest BLASTn matches, the taxonomic reliability of reference records, and ITS-based phylogenetic placement, with priority given to ex-type or authenticated reference sequences when available. Isolates showing <95% ITS identity to their closest reference sequences were retained as low-similarity phylotypes rather than being assigned to described species (Table S4). Because only ITS sequences were used to organize the full collection, these assignments were treated as a genus/phylotype-level organizational framework rather than as formal species-level identifications, especially for species-rich genera such as *Aspergillus*, *Penicillium*, *Cladosporium*, and *Talaromyces*. Formal species-level resolution of isolates in these genera will require genus-appropriate multilocus analyses, such as β-tubulin (BenA), calmodulin (CaM), the second-largest subunit of RNA polymerase II (RPB2) and/or other loci, together with colony morphology, micromorphological characterization, and comparison with ex-type or authenticated reference material. Sequences were deposited in GenBank under accession numbers PQ302328–PQ302480 and PX857774–PX857779.

Phylogenetic placement of the 159 culturable isolates was performed by maximum-likelihood inference of ITS sequences. The full dataset (159 DSF isolates, 65 GenBank reference sequences, and *Allomyces anomalus* ATCC 10982 [HQ888721] as outgroup) was aligned with MAFFT v7.526 (--auto) [26]. An initial maximum-likelihood tree was inferred in IQ-TREE v3.1.2 [27] under the best-fit substitution model (GTR+R6) selected by ModelFinder [28] under the Bayesian information criterion (BIC), with branch support assessed by 1000 ultrafast bootstrap replicates [29] and 1000 Shimodaira–Hasegawa-like approximate likelihood ratio test (SH-aLRT) replicates. The full tree is provided as Figure S1.

A representative dataset was then assembled for the main figure based on the closest-match taxonomic assignments and the topology of the full tree, retaining at least one isolate per closest-match taxonomic unit and additional representatives for heterogeneous lineages, distinct subclades, long-branched isolates, and isolates with low similarity to reference sequences; densely sampled terminal lineages were represented by selected isolates. The final dataset (85 DSF isolates, 65 references, and the outgroup) was realigned and reanalyzed under identical IQ-TREE settings, with the best-fit model selected as GTR+R5. The resulting tree was visualized and annotated in iTOL v6 [30].

### 2.5. In Vitro Antagonism Assay: Dual Culture

Twenty-three representative isolates were selected for primary antagonism screening based on phylogenetic placement, taxonomic representation, and the inclusion of frequently recovered metabolite-producing genera, particularly *Penicillium* and *Aspergillus* [13,14]. The assay targeted five phytopathogenic strains: three strains of *C. gloeosporioides*—CH008, isolated from stylo (*Stylosanthes* sp.); 171-1, isolated from mango; and RC178, isolated from rubber tree—together with *G. pseudoferreum* GP030 and *P. noxius* Pn006, all of which were provided by the Chinese Academy of Tropical Agricultural Sciences. The dual-culture assay was performed as described by Al-Rashdi et al. [31] with modifications. A 5-mm mycelial plug of a pathogen was placed at the center of a 9-cm PDA plate supplemented with streptomycin sulfate (100 μg/mL), and four 5-mm plugs of a sediment-derived fungal isolate were inoculated at points equidistant (2.5 cm) from the center. Control plates contained only the pathogen. Plates were incubated at 28 °C until pathogen mycelial growth on the control reached the plate edge or had stopped advancing for three consecutive days. Pathogen colony area was measured from photographs in ImageJ v1.50i [32], and the percentage of mycelial growth inhibition (MGI) was calculated as MGI (%) = [(Ac − At)/Ac] × 100, where Ac and At are the pathogen growth areas on the control and treatment plates, respectively. Each isolate–pathogen combination was tested using three independently prepared assay plates.

### 2.6. Culture Filtrate Assay

Nine isolates were selected for the culture filtrate assay based on broad-spectrum antagonism in the dual-culture assay and taxonomic representativeness, including one low-similarity phylotype. Their closest-match assignments are provided in Table S4. Each isolate was cultured in 50 mL of potato dextrose broth in a 250-mL flask at 28 °C and 180 rpm for 7 days [33]. The culture was centrifuged at 7830 rpm for 10 min, and the supernatant was filter-sterilized through a 0.22 μm membrane. The filtrate was incorporated into autoclaved PDA at 20% (*v*/*v*) by replacing an equivalent volume of distilled water; control plates received the same volume of sterile distilled water in place of filtrate. This design was used as a preliminary culture-filtrate screening assay and did not include a matched uninoculated-broth control. All plates contained streptomycin sulfate (100 μg/mL). A 5-mm mycelial plug of the target pathogen was placed at the center of each plate, incubated at 28 °C, and MGI was determined as in Section 2.5 using three independently prepared assay plates for each filtrate–pathogen combination.

### 2.7. Volatile Organic Compound Assay

The antifungal activity of VOCs produced by the nine isolates was evaluated using the dual-compartment Petri dish method adapted from previous studies [34]. This assay was used as a preliminary screen for volatile-mediated effects on pathogen mycelial growth rather than for chemical identification of individual VOCs. For each test, two 90-mm PDA plates supplemented with streptomycin sulfate (100 μg/mL) were prepared. One plate was centrally inoculated with a 5-mm plug of the target pathogen; the companion plate was either centrally inoculated with a plug of a sediment-derived fungal isolate or left uninoculated as the control. The two plates were joined base-to-base and sealed with three layers of Parafilm to prevent air exchange [35]. Assemblies were incubated at 28 °C and MGI was determined as in Section 2.5 using three independently prepared sealed-plate assemblies for each antagonist–pathogen combination.

### 2.8. Data Analysis and Statistics

Culturable fungal diversity was assessed in PAST v4.17 [36] using observed taxonomic units, Shannon index (H′), Simpson index (1−D), Pielou’s evenness (J), and Chao1. Chao1 was calculated using the bias-corrected estimator. Because isolates were purified after morphotype-based grouping rather than exhaustive colony counting, these indices were used to describe the recovered isolate collection and should not be interpreted as quantitative estimates of the original in situ fungal diversity or abundance of the sediment materials. For alpha- and beta-diversity analyses, isolates were grouped at the genus/phylotype level. Isolates with ≥95% ITS identity to their closest GenBank reference sequences were grouped according to their closest-match genera, whereas isolates with <95% ITS identity were treated as separate low-similarity phylotypes (LSP_01–LSP_22). This resulted in 57 taxonomic units, comprising 35 closest-match genus-level groups and 22 low-similarity phylotypes.

For visualization of taxonomic composition, the 22 low-similarity phylotypes were pooled as “Low-similarity phylotypes”; this pooling was not applied to alpha- or beta-diversity calculations. Beta diversity was assessed using a Bray–Curtis distance matrix calculated in PAST v4.17 [36], and principal coordinate analysis (PCoA) plots were generated in ImageGP 2 (https://www.bic.ac.cn/ImageGP/; accessed on 22 July 2026) [37]. Taxonomic composition plots were also generated in ImageGP 2 [37]. For the three in vitro antagonism assays, including dual culture, culture filtrate, and VOC assays, MGI values are reported as the mean ± standard deviation of three independently prepared assay units for each treatment combination, as described in Section 2.5, Section 2.6 and Section 2.7. For each pathogen, differences in MGI among isolates were tested by one-way analysis of variance (ANOVA) followed by Tukey’s honestly significant difference (HSD) post hoc test in IBM SPSS Statistics v26.0 (IBM Corp., Armonk, NY, USA) [38], with *p* < 0.05 considered statistically significant.

## 3. Results

### 3.1. Taxonomic Composition and Phylogenetic Placement of Culturable Isolates

A total of 159 culturable fungal isolates were recovered from the four deep-sea sediment materials using the 12 isolation protocols described above (Tables S2, S4, and S5). Based on ITS closest-match analysis and preliminary phylogenetic placement, the 159 isolates were placed within three phyla: Ascomycota, Basidiomycota, and Mucoromycota. Ascomycota was predominant, comprising 128 isolates, followed by Basidiomycota with 30 isolates and Mucoromycota with one isolate. Among the recovered isolates, 122 showed ≥95% ITS identity to their closest GenBank reference sequences and were provisionally assigned to 35 closest-match genus-level groups distributed across 17 orders. The remaining 37 isolates showed <95% ITS identity and were resolved into 22 operational low-similarity phylotypes (LSP_01–LSP_22) rather than being assigned to described species. A detailed genus/phylotype-level composition table is provided as Table S5, and the main taxonomic patterns are summarized below.

The 37 low-similarity isolates represented 23.3% of the recovered collection and were recovered from all four sediment materials. They were placed across Ascomycota, Basidiomycota, and Mucoromycota in the ITS tree and are therefore regarded as priority targets for future multilocus and morphology-based taxonomic study. However, because low ITS similarity may reflect taxonomic divergence, incomplete reference database coverage, or marker-specific resolution limits, we treated these isolates as operational low-similarity phylotypes and did not infer species novelty from ITS data alone.

**Figure 2:**
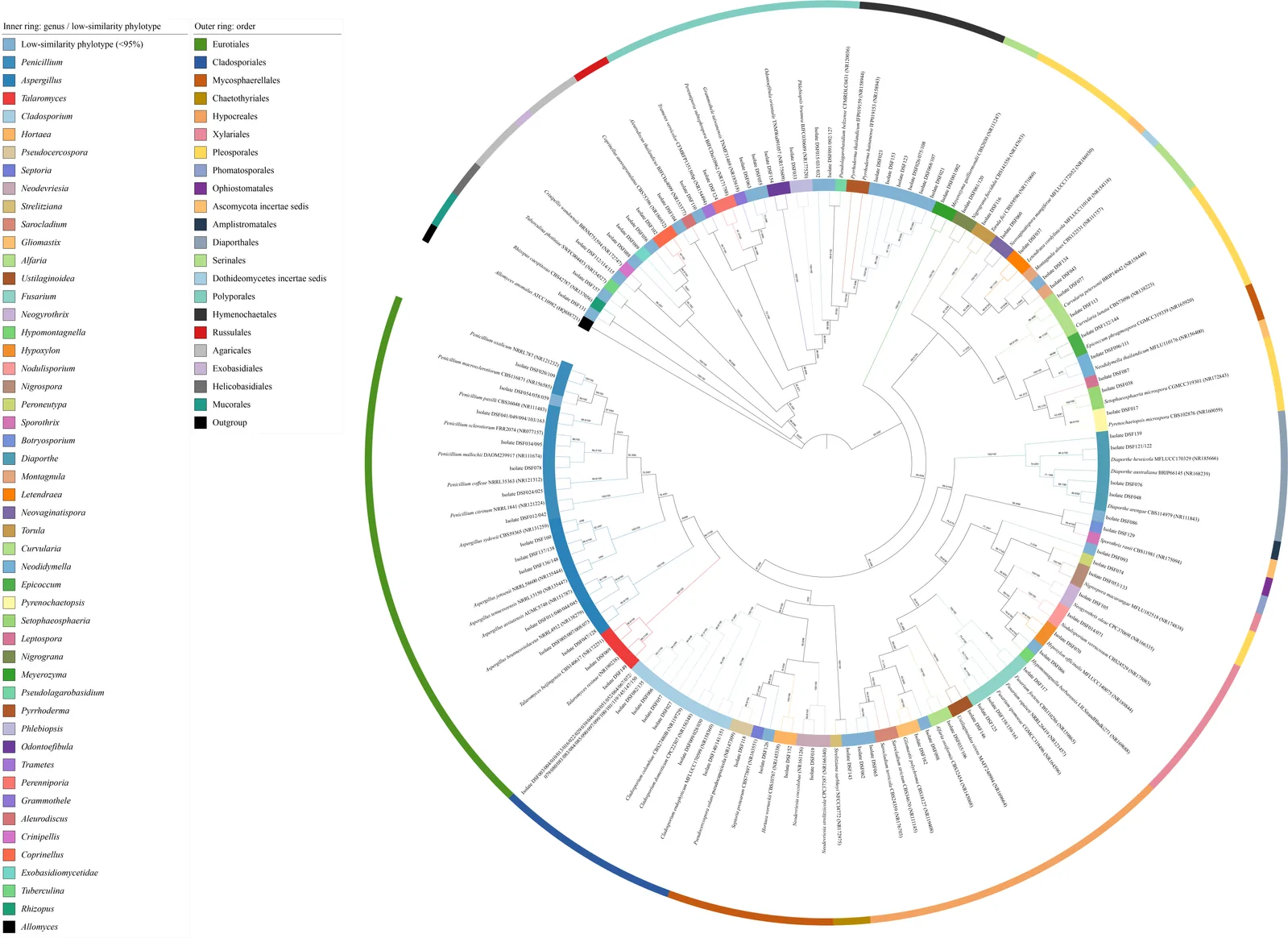
Maximum-likelihood phylogeny inferred from ITS sequences of representative culturable fungal isolates recovered from deep-sea sediments and selected GenBank reference sequences. Branch labels indicate SH-aLRT support/ultrafast bootstrap support values. The inner and outer rings denote genus-level group or low-similarity phylotype and order-level placement, respectively. *Allomyces anomalus* ATCC 10982 (HQ888721) was used as the outgroup. This ITS-based phylogeny should be interpreted as a preliminary placement framework rather than as robust species-level phylogenetic resolution.

The maximum-likelihood phylogeny based on the representative ITS dataset resolved the recovered isolates within major fungal lineages consistent with their closest-match assignments (Figure 2). Eurotiales contained the main *Aspergillus*, *Penicillium*, and *Talaromyces* lineages, whereas *Cladosporium* isolates formed the most densely sampled lineage within Cladosporiales. The 22 low-similarity phylotypes were placed within all three recovered phyla, with 11 phylotypes in Basidiomycota, 10 in Ascomycota, and one (LSP_14) in Mucoromycota, further supporting their prioritization for future taxonomic refinement rather than formal species-level treatment in the present study. At the genus level, *Cladosporium* was the most frequently recovered group, represented by 41 isolates (25.8%), followed by *Penicillium* with 17 isolates (10.7%) and *Aspergillus* with 15 isolates (9.4%). Most other assigned genera were represented by five or fewer isolates, indicating a recovered collection characterized by a few abundant genera and many low-frequency taxa.

### 3.2. Distribution of Culturable Isolates Among Sediment Materials and Isolation Protocols

**Figure 3:**
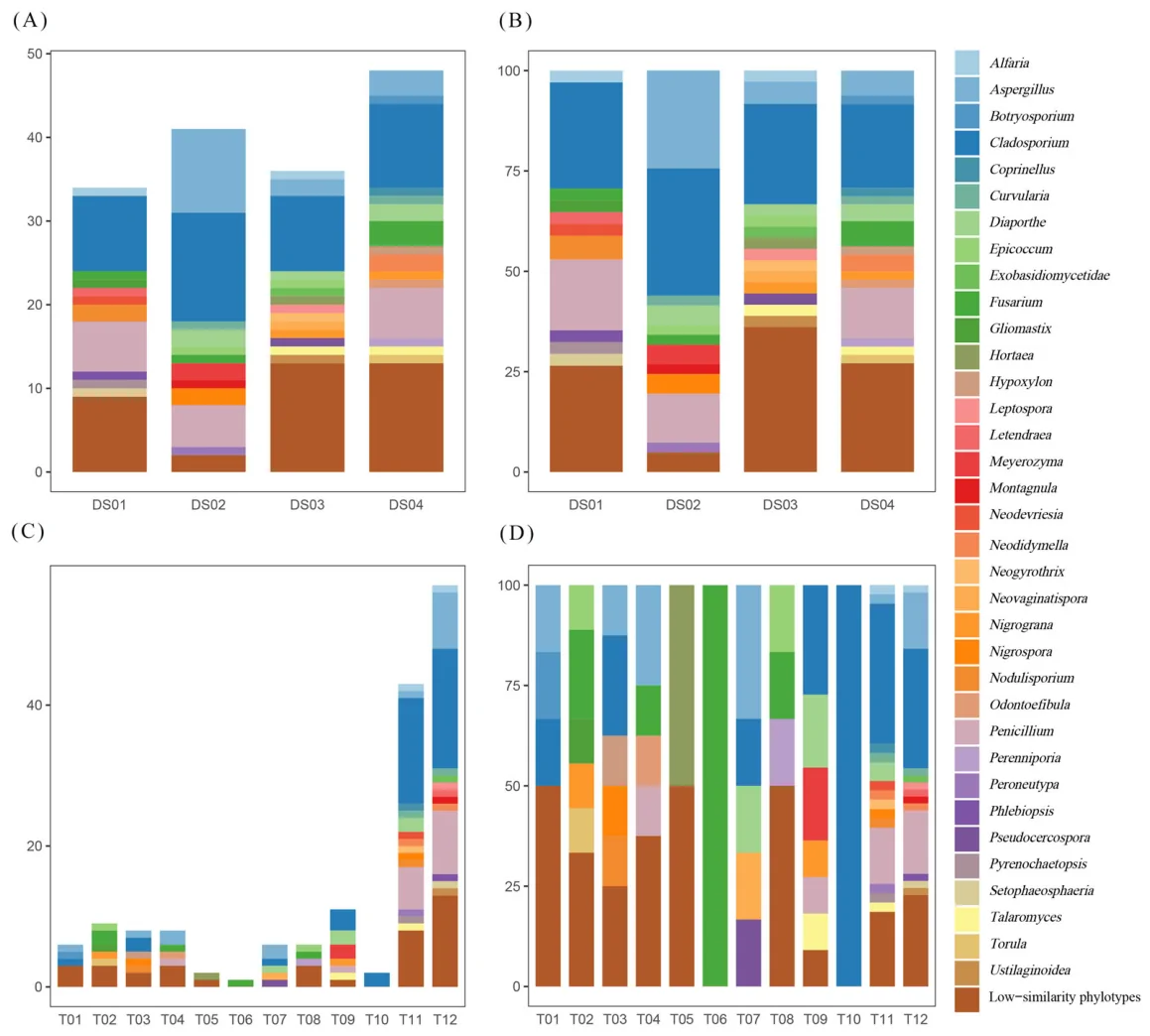
Distribution of culturable fungal isolates among sediment materials and isolation protocols. (**A**,**B**) Genus-level composition of isolates recovered from the four sediment materials. (**C**,**D**) Genus-level composition of isolates recovered using the 12 isolation protocols. Panels (**A**,**C**) show the number of isolates, whereas panels (**B**,**D**) show relative abundance. Low-similarity phylotypes were pooled for visualization.

The number and composition of recovered isolates varied among the four operational sediment materials (Figure 3A,B), which represented three sampling units because DS03 and DS04 were derived from different sediment fractions at the same South China Sea station. DS04 yielded the largest number of isolates, with 48 isolates representing 26 taxa, including 15 closest-match genera and 11 low-similarity phylotypes, and accounted for 30.2% of the total collection. DS02 yielded 41 isolates representing 13 taxa and accounted for 25.8% of the total collection, DS03 yielded 36 isolates representing 22 taxa (22.6%), and DS01 yielded 34 isolates representing 15 taxa (21.4%). *Cladosporium* was recovered from all four materials and was among the most abundant assigned genera in each sediment material, with within-material frequencies ranging from 20.8% to 31.7%. *Penicillium* was detected in DS01, DS02, and DS04, whereas *Aspergillus* was detected in DS02, DS03, and DS04 but not in DS01. *Aspergillus* was particularly frequent in DS02, where it accounted for 24.4% of the recovered isolates. The within-material proportion of low-similarity isolates was 26.5% in DS01, 4.9% in DS02, 36.1% in DS03, and 27.1% in DS04.

In addition to the dominant genera, many assigned genera were rare or material-restricted. Twenty-one closest-match genera were represented by a single isolate in the full collection, and several genera were detected in only one sediment material, with seven material-restricted genera in DS01, four in DS02, seven in DS03, and seven in DS04. These patterns indicate that the four sediment materials differed not only in dominant taxa but also in their rare and material-restricted components.

Fungal recovery also varied among isolation protocols (Figure 3C,D). T12, corresponding to plate stamping on GPY medium, yielded 57 isolates representing 23 taxa, including 13 closest-match genera and 10 low-similarity phylotypes, and accounted for 35.8% of the total collection. T11, corresponding to plate stamping on Fungus No. II medium, yielded 43 isolates representing 20 taxa, including 15 closest-match genera and five low-similarity phylotypes, and accounted for 27.0%. Together, the two plate-stamping protocols recovered 100 isolates, representing 62.9% of the collection. By comparison, the four untreated 10^−1^ dilution-plating protocols (T01–T04) recovered 31 isolates representing 22 taxa, whereas the four untreated 10^−2^ dilution-plating protocols (T05–T08) recovered 15 isolates representing 13 taxa. EDTA pretreatment on MEA (T09 and T10) recovered 13 isolates representing seven taxa, which was similar in magnitude to the recovery obtained with the corresponding untreated MEA dilution protocols.

Overall, the recovered culturable fungal collection varied among sediment materials and isolation protocols under the present cultivation framework. The two plate-stamping protocols recovered a large and compositionally distinct subset of isolates, including taxa not fully represented by dilution-based protocols. Because plate stamping and dilution plating differed in inoculum preparation, sediment transfer, and propagule deposition, these patterns are best interpreted as protocol-dependent differences in recovered culturable fractions rather than as a direct quantitative comparison of isolation efficiency.

### 3.3. Alpha and Beta Diversity of the Recovered Culturable Assemblages

Alpha-diversity indices were calculated using the 57 genus/phylotype-level taxonomic units defined in Section 2.8 to compare the recovered culturable assemblages among operational sediment materials and isolation protocols (Table 1 and Table 2). These indices describe the morphotype-selected recovered isolate collection rather than the original in situ fungal diversity or abundance of the sediment materials. Among the four sediment materials, DS04 showed the highest observed richness, with 26 taxa, and also had the highest Shannon index (*H*′ = 2.904) and Simpson index (*1* − *D* = 0.918). DS03 followed, with 22 observed taxa and a Shannon index of 2.774. DS01 and DS02 showed lower observed richness, with 15 and 13 taxa, respectively. Pielou’s evenness was relatively high across the four sediment materials (*J* = 0.796–0.897), indicating that the recovered taxa were not strongly dominated by a single taxon. Chao1 estimates exceeded the observed taxon numbers in all four sediment materials and were highest in DS04 (83.00) and DS03 (67.33), suggesting that additional culturable diversity may remain unsampled under the present isolation framework, particularly given the limited number of isolates recovered from each sediment material.

PCoA based on the Bray–Curtis distance matrix at the 57-taxon resolution provided a visual summary of compositional dissimilarities among the four operational sediment materials (Figure S2A). The first two principal coordinates explained 78.0% of the total variation, with PCo1 and PCo2 accounting for 44.2% and 33.8%, respectively. DS03 plotted apart from the other materials along PCo1, corresponding to its distinct recovered taxon composition, including a high representation of low-similarity phylotypes. DS01 was offset from the other three materials along PCo2, whereas DS02 and DS04 plotted closer to each other along PCo1. These patterns should be interpreted as descriptive differences among recovered isolate collections rather than as replicated tests of sediment-source effects.

Alpha-diversity indices also varied among isolation protocols (Table 2). The observed number of taxa per protocol ranged from one in T06 and T10 to 23 in T12. The two plate-stamping protocols yielded the largest recovered taxon numbers, with 20 taxa in T11 and 23 taxa in T12. T12 also showed the highest Shannon index among all protocols (*H′* = 2.486), followed by T11 (*H′* = 2.405). Together, the two plate-stamping protocols captured 35 taxa, corresponding to 61.4% of the 57 taxa in the full collection. By comparison, the four untreated 10^−1^ dilution-plating protocols (T01–T04) collectively recovered 22 taxa. Chao1 estimates exceeded observed taxon numbers for most protocols and were highest in T11 (80.00) and T12 (57.00). Elevated Chao1 estimates were also observed for some protocols with low isolate numbers, such as T01 and T08, indicating that richness estimates based on sparse isolate counts should be interpreted cautiously.

PCoA based on the Bray–Curtis distance matrix at the 57-taxon resolution further showed compositional differences among isolation protocols (Figure S2B). The first two principal coordinates explained 44.6% of the total variation, with PCo1 and PCo2 accounting for 27.7% and 16.9%, respectively. The two plate-stamping protocols, T11 and T12, plotted close to each other and were separated from most dilution-based protocols, consistent with their recovery of a relatively large and partly shared subset of isolates. In contrast, the dilution-based protocols were more dispersed, reflecting differences in the subsets of taxa recovered by individual dilution, medium, and pretreatment combinations. These patterns were consistent with the recovery of a compositionally distinct subset of culturable fungi by plate stamping relative to dilution-based protocols under the present non-equivalent inoculation conditions.

### 3.4. In Vitro Antagonism Assessed by Dual-Culture Assay

**Figure 4:**
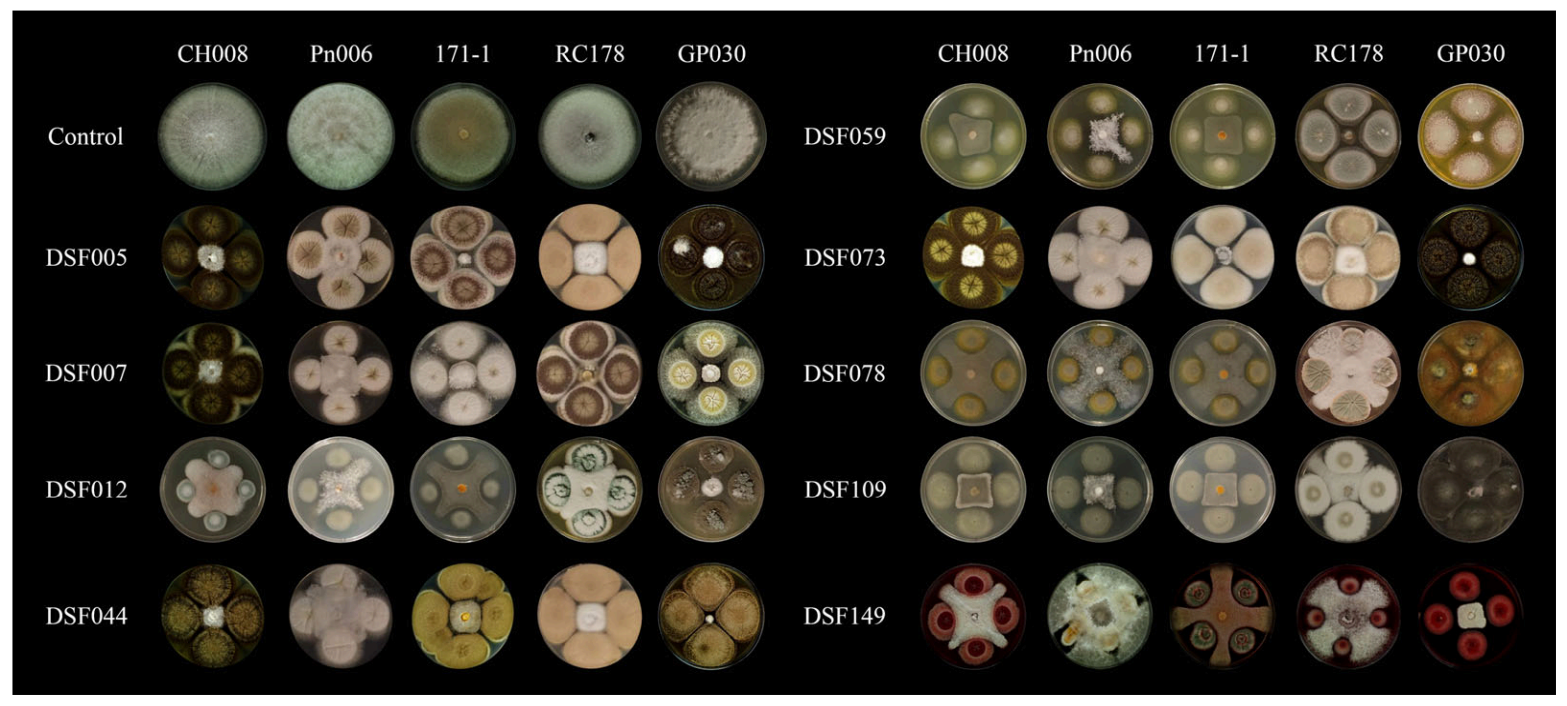
Dual-culture colony phenotypes of five phytopathogenic fungal strains confronted with nine representative deep-sea sediment-derived fungal isolates selected for downstream culture filtrate and VOC assays. Pathogen-only plates served as controls. The complete set of colony photographs for the remaining tested isolates is provided in Figure S5. CH008, 171-1, and RC178 are strains of *Colletotrichum gloeosporioides*; Pn006 represents *Phellinus noxius*; and GP030 represents *Ganoderma pseudoferreum*.

Based on the phylogenetic results and the high representation of *Penicillium* and *Aspergillus* in the recovered collection, 23 representative isolates were selected for preliminary phenotype-based antagonism screening against five phytopathogenic strains. The selected isolates mainly had closest affinities to *Penicillium*, *Aspergillus*, and *Talaromyces*, including one low-similarity phylotype with closest affinity to *Penicillium*. The dual-culture assay revealed clear isolate- and pathogen-dependent inhibition patterns (Table 3). Representative dual-culture plate phenotypes for the nine isolates selected for downstream culture filtrate and VOC assays are shown in Figure 4, and plate phenotypes for the remaining tested isolates are provided in Figure S5.

The three strains of *C. gloeosporioides* differed in their responses to the tested isolates. Inhibition of CH008 ranged from 0.00 ± 0.00% to 93.14 ± 0.91%, with the highest value recorded for DSF044, followed by DSF073, DSF007, and DSF005. For strain 171-1, inhibition ranged from 0.00 ± 0.00% to 96.65 ± 0.80%, and the strongest activity was observed for DSF005, DSF007, DSF073, and DSF044. For RC178, inhibition ranged from 0.00 ± 0.00% to 95.57 ± 0.00%, with DSF007 producing the highest inhibition, followed by DSF059, DSF005, DSF044, and DSF073.

The two basidiomycete pathogens also showed distinct response profiles. Against *P. noxius* Pn006, inhibition ranged from 0.00 ± 0.00% to 84.58 ± 0.82%, with DSF059, DSF109, DSF005, DSF012, and DSF007 among the most active isolates. *G. pseudoferreum* GP030 was generally more sensitive to the tested isolates, with inhibition ranging from 5.82 ± 7.70% to 98.74 ± 0.00%. DSF044 produced the highest inhibition against GP030, and several other isolates, including DSF078, DSF007, DSF109, DSF012, DSF095, DSF073, and DSF103, also showed inhibition values above 90%.

Overall, DSF005, DSF007, DSF012, DSF044, DSF059, DSF073, DSF078, and DSF109 showed relatively strong inhibition across multiple pathogen strains. In contrast, DSF047, DSF137, DSF138, DSF148, and DSF160 showed weak or pathogen-limited inhibition against most tested pathogens, although several of these isolates still inhibited GP030 to some extent. These results indicate that antagonistic activity varied markedly among isolate–pathogen combinations rather than being uniform across the tested fungal collection, supporting the use of this assay as a preliminary screen for prioritizing isolates for further study.

### 3.5. Antifungal Activity of Culture Filtrates

Sterile culture filtrates from nine selected isolates were tested to determine whether extracellular culture products could reproduce part of the inhibition observed in dual culture. The assay showed isolate- and pathogen-dependent effects, with MGI values ranging from −35.21 ± 2.59% to 84.09 ± 4.62% (Table 4). Compared with dual culture, the filtrate assay produced more variable and less consistently inhibitory responses, indicating that filtrate-associated effects accounted for only part of the antagonism observed during direct fungal confrontation.

Among the three *C. gloeosporioides* strains, culture filtrate-mediated inhibition differed among CH008, 171-1, and RC178. Against CH008, inhibition ranged from 0.00 ± 0.00% to 39.42 ± 4.40%, with the largest values recorded for DSF012, DSF149, DSF005, DSF044, and DSF109. For strain 171-1, inhibition was generally weak or absent for most filtrates; DSF012 showed the highest inhibition value of 40.31 ± 6.88%, whereas DSF078 and DSF044 produced lower but detectable inhibition and DSF005 yielded a slight negative MGI value. Against RC178, the strongest inhibition was observed for DSF005, followed by DSF109 and DSF012, whereas the filtrates of DSF044, DSF073, and DSF078 showed no detectable inhibition.

The two basidiomycete pathogens also differed in their responses. Against *P. noxius* Pn006, DSF044 showed the strongest inhibition, reaching 84.09 ± 4.62%, followed by DSF149, DSF109, and DSF012. By contrast, DSF005, DSF073, DSF078, and DSF059 showed little or no inhibition against Pn006. Against *G. pseudoferreum* GP030, the strongest inhibition was observed for DSF012, followed by DSF149, DSF005, DSF073, and DSF007. Notably, the filtrates of DSF044, DSF059, and DSF109 produced negative MGI values against GP030, indicating that pathogen colony growth was greater than that of the corresponding control under these assay conditions.

The colony photographs were consistent with the quantitative measurements and showed marked reductions in colony area in several isolate–pathogen combinations, particularly DSF044 against Pn006 and DSF012 against GP030 (Figure S3). Together, these results indicate that culture filtrate-mediated effects were highly specific to particular isolate–pathogen combinations under the present preliminary screening conditions. The discrepancy between the dual-culture and culture filtrate assays further suggests that direct interaction, competition, and other non-filtrate-mediated effects may also contribute to the antagonistic activity of the selected isolates.

### 3.6. Antifungal Activity of Fungal Volatile Organic Compounds

Volatile-mediated effects of the nine selected isolates were evaluated against the five phytopathogenic strains using the sealed-plate assay. This assay was used as a preliminary screen for growth effects mediated by volatile emissions, without chemical identification of individual VOCs. The assay revealed clear isolate- and pathogen-dependent responses, with MGI values ranging from −25.74 ± 0.00% to 63.25 ± 7.13% (Table 5). In the VOC assay, inhibition was most consistently observed for CH008 and GP030, whereas responses of Pn006 and RC178 were weaker or more variable.

Among the three *C. gloeosporioides* strains, CH008 was inhibited by VOCs from all nine isolates, with inhibition values ranging from 10.72 ± 6.17% to 50.80 ± 1.36%. The highest inhibition was observed for DSF073 and DSF044, followed by DSF109, DSF059, and DSF007. For strain 171-1, VOC-mediated inhibition was more variable, ranging from 0.00 ± 0.00% to 51.17 ± 10.43%. DSF005 and DSF109 showed the strongest inhibition, whereas DSF007 showed no detectable inhibition. In contrast, RC178 showed limited sensitivity to most VOC treatments. DSF149 showed the highest inhibition against RC178, followed by DSF109 and DSF012, whereas VOC exposure from DSF005, DSF007, DSF044, and DSF073 resulted in negative MGI values for RC178, indicating larger pathogen colonies than the corresponding control under the present assay conditions.

The two basidiomycete pathogens responded differently to fungal VOCs. Against *P. noxius* Pn006, inhibition was generally weak, with most values close to zero; DSF109 produced the highest inhibition, followed by DSF059, DSF012, DSF078, and DSF005. By contrast, *G. pseudoferreum* GP030 was more responsive to VOC exposure. DSF007 showed the strongest inhibition against GP030, followed by DSF073, DSF044, DSF059, and DSF005. VOCs from DSF078 and DSF109 showed little or no inhibition against GP030.

The colony photographs were consistent with the quantitative MGI values and showed that volatile-mediated effects varied strongly among isolate–pathogen combinations (Figure S4). Together, these results indicate that VOC-mediated activity was not uniformly inhibitory across the tested isolates and pathogens. The occurrence of negative MGI values further indicates that exposure to fungal volatiles can result in either reduced or increased pathogen colony growth under the present sealed-plate screening conditions, depending on the isolate–pathogen combination.

Across the three assay formats, the nine selected isolates showed partially discordant antagonistic profiles. For example, DSF044 showed very strong inhibition against *G. pseudoferreum* GP030 in dual culture, but its culture filtrate produced a negative MGI value against the same pathogen. Similarly, DSF109 showed strong dual-culture inhibition against GP030 but showed negative filtrate-associated activity and only weak VOC-mediated inhibition against this pathogen. In contrast, DSF012 showed strong filtrate-associated inhibition against GP030, whereas several isolates with strong dual-culture activity showed weaker or pathogen-specific responses in the filtrate and VOC assays. These contrasting patterns indicate that direct confrontation, filtrate-associated effects, and volatile-mediated effects captured different components of the antagonistic interaction. Thus, assay-format-dependent variation should be considered when prioritizing isolates for downstream mechanistic and biocontrol-oriented studies.

## 4. Discussion

The present study establishes an ITS-organized living fungal collection recovered from deep-sea sediment materials and links this resource to protocol-resolved recovery patterns and multi-format antagonistic screening. The collection includes recurrently recovered filamentous genera, low-similarity ITS phylotypes preserved as living cultures, and isolates displaying assay-dependent inhibition of phytopathogenic fungi. Together, these features demonstrate the value of combining complementary cultivation protocols, conservative molecular placement, and phenotype-based screening to prioritize deep-sea sediment-derived fungi for subsequent taxonomic, chemical, and biocontrol-oriented investigations.

The predominance of Ascomycota and the frequent recovery of *Cladosporium*, *Penicillium*, and *Aspergillus* are consistent with culture-dependent studies from geographically distinct deep-sea and deep-marine habitats, including the Central Indian Basin, the South China Sea, the South Atlantic Ocean, the Okinawa Trough, hydrothermal vent ecosystems, and deep-subseafloor sediments [5,6,7,39,40,41]. The recurrence of these genera likely reflects both their biological characteristics and their ability to grow under commonly used laboratory conditions. Efficient sporulation, broad dispersal, stress tolerance, and rapid growth may favor their representation in culture-dependent collections. At the same time, *Aspergillus*, *Penicillium*, *Cladosporium*, and *Talaromyces* include metabolically productive and experimentally tractable lineages that remain important sources of structurally diverse fungal secondary metabolites [13,14]. Their recovery therefore contributes both taxonomic breadth and accessible material for phenotype-guided investigation.

The observed recovery patterns further emphasize the importance of cultivation design. Plate stamping recovered a large and compositionally distinct subset of isolates relative to dilution-based protocols. Direct transfer of sediment particles to agar may retain particle-associated propagules or inoculum structures that are diluted, dispersed, or competitively disadvantaged during suspension-based plating, consistent with reports of fungi associated with deep-sea sediment aggregates [18]. More broadly, deep-sea cultivation studies have shown that incubation and inoculation conditions can strongly influence the fraction of sediment-associated microorganisms recovered in culture [42]. Because plate stamping and dilution plating differed in sediment transfer, inoculum preparation, and propagule deposition, the present results indicate protocol-dependent access to different culturable fractions rather than quantitatively comparable isolation efficiencies. The antibiotic cocktail facilitated fungal recovery by suppressing bacterial overgrowth, although its effects were not examined independently. Antibiotic supplementation may have altered bacterial–fungal interactions or favored fungi capable of growth under antibiotic-amended conditions. In addition, because isolates were retained through morphotype-based selection rather than exhaustive colony counting, the diversity indices describe the recovered living collection rather than the abundance or diversity of the original sediment fungal community.

Low-similarity ITS phylotypes represent an important component of this collection. Sequence-based studies have repeatedly detected unknown, uncultured, or poorly resolved fungal lineages in deep-sea sediments [1,2,3,43,44]. Unlike sequence-only records, the phylotypes recovered here are available as living cultures for morphological, physiological, chemical, and multilocus investigation [13,14,45]. Their low ITS similarity may reflect taxonomic divergence, incomplete database representation, or marker-specific resolution limits; accordingly, they are best regarded as priority targets for taxonomic refinement. The same caution applies to closest-match names within *Aspergillus*, *Penicillium*, *Cladosporium*, and *Talaromyces*. ITS provides an effective framework for organizing a broad culture collection, but reliable species-level identification in these genera requires genus-appropriate multilocus markers, morphological and micromorphological characterization, and comparison with ex-type or authenticated reference material [8,9,10,11,12].

The ecological status of fungi recovered from deep-sea sediments remains unresolved. Successful cultivation demonstrates that viable fungal propagules or cells were present in the sediment materials, but does not establish whether they were actively growing, adapted residents, dormant propagules, or fungi transported from terrestrial or shallow-water environments. Marine fungal ecology studies have similarly emphasized the difficulty of distinguishing resident fungi from transient or dispersed propagules [46,47]. The recovered collection may therefore include both sediment-associated fungi capable of persistence under marine conditions and allochthonous or dormant propagules introduced through water movement, particle flux, terrestrial runoff, atmospheric dispersal, or related pathways. The antagonism assays were conducted at 28 °C to support phytopathogen growth and assess agricultural screening potential. Experimental evidence indicates that deep-sea-relevant conditions, including elevated hydrostatic pressure, can alter fungal development and secondary metabolite production [48,49], while cultivation conditions, including temperature, can influence metabolite profiles and bioactivity [17]. These findings support further testing of priority isolates under lower temperature, oligotrophic media, salinity gradients, sediment-particle-associated incubation, and elevated pressure.

The antagonistic assays add a functional dimension to the ITS-organized collection and show that taxonomic placement alone does not predict inhibitory activity. The partially discordant profiles obtained from dual-culture, culture filtrate, and VOC assays revealed distinct assay-format-dependent phenotypes across direct-contact and non-contact interaction contexts. Dual culture integrates direct hyphal confrontation, spatial and nutrient competition, diffusible products, interaction-induced responses, and volatile-mediated effects. Culture filtrate and sealed-plate VOC assays, by contrast, examine selected non-contact components under defined experimental conditions. Consequently, strong dual-culture inhibition accompanied by weak filtrate or VOC activity may indicate a greater contribution from direct competition or interaction-zone processes, whereas reproducible filtrate- or VOC-associated inhibition identifies candidates for metabolite- or volatile-focused analysis. This assay-format-dependent divergence is consistent with the chemical and mechanistic diversity reported for marine microbial natural products with potential agricultural applications [50,51] and provides a practical basis for prioritizing isolates for different downstream investigations. The strain- and pathogen-dependent VOC responses observed here are consistent with the diverse ecological and biotechnological effects reported for fungal volatiles [52,53,54,55]. Accordingly, the present assay design distinguishes contact-associated, filtrate-associated, and volatile-mediated phenotypes, but does not resolve the relative contributions of hydrolytic enzymes, nutrient competition, individual metabolites, or specific biosynthetic pathways.

The culture filtrate results should also be considered in relation to the assay matrix. Filtrate-amended PDA was compared with water-amended PDA rather than with PDA containing uninoculated PDB processed in parallel. Residual nutrients, pH changes, or other broth-derived components may therefore have influenced pathogen growth and contributed to some of the negative MGI values. The assay nevertheless provides a useful preliminary screen for filtrate-associated effects, provided that active components are subsequently evaluated using matched controls, purification, and bioassay-guided analysis. Similarly, the sealed-plate assay demonstrates volatile-mediated effects on pathogen growth but does not identify the responsible compounds. Further work should combine matched controls with chemical profiling and bioassay-guided fractionation to identify filtrate- and VOC-associated metabolites [50,51].

Several considerations define the scope of the present study. The four sediment materials represented three sampling units, with DS03 and DS04 originating from different fractions at the same South China Sea station; the compositional patterns therefore describe operational recovery profiles rather than replicated tests of biogeographic, depth-related, or sediment-layer effects. ITS-based placement organizes the collection at the genus/phylotype level, while the in vitro assays provide phenotype-based prioritization under laboratory conditions. Together, these considerations define the scope of this resource-establishment and screening study and point directly to the next priorities. Low-similarity phylotypes and high-priority antagonistic isolates should undergo genus-appropriate multilocus analysis and detailed morphological characterization. Selected isolates should also be tested under marine sediment-relevant cultivation conditions and examined through time-resolved interaction assays, microscopy of confrontation zones, and pathogen-response analyses. For the most informative candidates, genome mining and biosynthetic pathway prediction [56,57], one strain–many compounds (OSMAC)-based cultivation [58], metabolomic or VOC-focused chemical profiling, molecular networking [59], and bioassay-guided fractionation can be integrated to connect inhibitory phenotypes with candidate pathways or chemical features. Subsequent evaluation of safety, host compatibility, colonization ability, formulation potential, and in planta efficacy will be required before biocontrol application [60]. This staged strategy provides a realistic route from living-resource recovery to taxonomic resolution, mechanistic interpretation, and targeted antifungal candidate development.

## 5. Conclusions

This study established an ITS-organized living fungal collection recovered from deep-sea sediment materials of the Mariana Trench slope region and the South China Sea. The use of 12 complementary isolation protocols yielded 159 culturable isolates placed within Ascomycota, Basidiomycota, and Mucoromycota, representing 35 closest-match genus-level groups and 22 low-similarity ITS phylotypes preserved as living cultures. Recovery patterns varied among sediment materials and protocols, with plate stamping capturing a large and compositionally distinct subset of isolates not fully represented by dilution-based methods, underscoring the value of methodological complementarity.

Representative isolates displayed assay-format-dependent in vitro antifungal activity against phytopathogenic fungi. The partial discordance among dual-culture, culture filtrate, and VOC assays revealed distinct phenotypes across direct-contact and non-contact interaction contexts. Together, the documented living collection and multi-format screening dataset provide a foundation for targeted taxonomic refinement, chemical characterization, mechanism-oriented investigation, and subsequent evaluation of biocontrol potential.

## Figures and Tables

**Table 1:** Alpha-diversity indices of culturable fungal assemblages recovered from four deep-sea sediment materials.

Sediment Material	No. of Isolates	Observed Taxonomic Units	Shannon (*H*′)	Simpson (*1*−*D*)	Pielou’s Evenness (*J*)	Chao1
DS01	34	15	2.328	0.865	0.860	37.50
DS02	41	13	2.041	0.814	0.796	18.25
DS03	36	22	2.774	0.904	0.897	67.33
DS04	48	26	2.904	0.918	0.891	83.00

**Table 2:** Alpha diversity indices of culturable fungal assemblages recovered using different isolation protocols.

Protocol	No. of Isolates	Observed Taxonomic Units	Shannon (*H′*)	Simpson (*1* − *D*)	Pielou’s Evenness (*J*)	Chao1
T01	6	6	1.792	0.833	1.000	21.00
T02	9	7	1.889	0.840	0.971	10.33
T03	8	7	1.906	0.844	0.980	14.50
T04	8	7	1.906	0.844	0.980	14.50
T05	2	2	0.693	0.500	1.000	3.00
T06	1	1	0.000	0.000	—	1.00
T07	6	5	1.561	0.778	0.970	8.00
T08	6	6	1.792	0.833	1.000	21.00
T09	11	7	1.846	0.826	0.949	9.00
T10	2	1	0.000	0.000	—	1.00
T11	43	20	2.405	0.839	0.803	80.00
T12	57	23	2.486	0.857	0.793	57.00

Note: Pielou’s evenness is undefined when only one taxon is observed and is denoted by an em dash.

**Table 3:** Dual-culture antagonistic activity of 23 representative deep-sea sediment-derived fungal isolates against five phytopathogenic fungal strains: three strains of *Colletotrichum gloeosporioides* CH008, 171-1, and RC178, *Phellinus noxius* Pn006, and *Ganoderma pseudoferreum* GP030.

Isolates	Closest GenBank BLASTn Match Based on ITS	ITS Similarity to Closest Match (%)	MGI (%)				
			CH008	Pn006	171-1	RC178	GP030
DSF005	*A. brunneoviolaceus* NRRL 4912 (NR_138279.1)	99.44	89.65 ± 0.87 ^a^	83.06 ± 2.15 ^a^	96.65 ± 0.80 ^a^	85.63 ± 0.89 ^a^	92.74 ± 2.27 ^ab^
DSF007	*A. brunneoviolaceus* NRRL 4912 (NR_138279.1)	98.08	90.40 ± 1.84 ^a^	79.09 ± 1.48 ^a^	89.95 ± 1.71 ^b^	95.57 ± 0.00 ^a^	95.83 ± 1.05 ^a^
DSF012	*P. citrinum* NRRL 1841 (NR_121224.1)	100.00	65.97 ± 3.29 ^c^	80.67 ± 0.85 ^a^	63.47 ± 0.68 ^e^	77.70 ± 1.92 ^a^	95.26 ± 1.55 ^a^
DSF024	*P. coffeae* NRRL 35363 (NR_121312.1)	99.06	5.53 ± 0.08 ^jk^	23.06 ± 0.70 ^ef^	7.43 ± 0.10 ^j^	10.54 ± 0.51 ^def^	89.45 ± 6.67 ^ab^
DSF025	*P. coffeae* NRRL 35363 (NR_121312.1)	99.06	14.03 ± 0.18 ^i^	5.01 ± 0.12 ^ijk^	9.24 ± 0.16 ^j^	23.39 ± 0.81 ^cd^	80.76 ± 2.78 ^bc^
DSF034	*P. sclerotiorum* FRR 2074 (NR_077157.1)	99.27	19.53 ± 0.24 ^gh^	11.70 ± 0.38 ^hi^	24.44 ± 0.80 ^i^	9.49 ± 0.74 ^def^	86.71 ± 9.83 ^abc^
DSF036	*P. coffeae* NRRL 35363 (NR_121312.1)	99.06	14.84 ± 0.43 ^hi^	4.28 ± 0.19 ^jk^	23.76 ± 0.78 ^i^	21.96 ± 1.03 ^cde^	52.99 ± 0.73 ^de^
DSF044	*A. assiutensis* AUMC 5748 (NR_151787.1)	100.00	93.14 ± 0.91 ^a^	60.83 ± 1.20 ^b^	84.06 ± 1.66 ^cd^	85.40 ± 0.89 ^a^	98.74 ± 0.00 ^a^
DSF047	*A. brunneoviolaceus* NRRL 4912 (NR_138279.1)	99.44	4.93 ± 0.55 ^jkl^	0.00 ± 0.00 ^k^	1.96 ± 3.39 ^kl^	11.25 ± 19.49 ^def^	5.82 ± 7.70 ^g^
DSF054	*P. macrosclerotiorum* CBS 116871 (NR_156585.1)	99.44	54.42 ± 5.89 ^d^	29.92 ± 5.18 ^de^	5.04 ± 4.36 ^jk^	58.06 ± 3.59 ^b^	60.14 ± 0.61 ^d^
DSF059	*P. macrosclerotiorum* CBS 116871 (NR_156585.1)	91.57	81.03 ± 0.84 ^b^	84.58 ± 0.82 ^a^	80.00 ± 0.84 ^d^	89.39 ± 7.10 ^a^	92.64 ± 3.50 ^ab^
DSF069	*T. beijingensis* CBS 140617 (NR_172251.1)	98.88	24.36 ± 0.28 ^g^	7.50 ± 0.00 ^ij^	55.52 ± 0.00 ^f^	24.67 ± 0.65 ^cd^	88.18 ± 1.20 ^abc^
DSF073	*A. brunneoviolaceus* NRRL 4912 (NR_138279.1)	98.90	91.32 ± 0.00 ^a^	66.74 ± 8.56 ^b^	87.73 ± 3.64 ^bc^	81.96 ± 2.53 ^a^	94.03 ± 2.36 ^a^
DSF078	*P. mallochii* DAOM 239917 (NR_111674.1)	99.06	43.00 ± 0.81 ^e^	47.98 ± 0.87 ^c^	56.92 ± 1.21 ^f^	32.49 ± 1.72 ^c^	96.87 ± 1.00 ^a^
DSF094	*P. paxilli* CBS 360.48 (NR_111483.1)	98.81	24.07 ± 0.61 ^g^	17.41 ± 0.38 ^fgh^	27.96 ± 0.80 ^i^	58.57 ± 1.71 ^b^	81.30 ± 1.88 ^bc^
DSF095	*P. sclerotiorum* FRR 2074 (NR_077157.1)	99.44	37.51 ± 1.51 ^f^	36.73 ± 1.12 ^d^	35.10 ± 0.39 ^h^	3.47 ± 0.27 ^ef^	94.12 ± 2.00 ^a^
DSF103	*P. paxilli* CBS 360.48 (NR_111483.1)	98.87	45.08 ± 2.15 ^e^	19.92 ± 0.63 ^fg^	26.90 ± 0.58 ^i^	7.56 ± 0.77 ^def^	94.03 ± 1.79 ^a^
DSF109	*P. oxalicum* NRRL 787 (NR_121232.1)	99.64	80.83 ± 0.80 ^b^	84.25 ± 2.39 ^a^	81.23 ± 0.81 ^d^	77.14 ± 3.65 ^a^	95.62 ± 1.72 ^a^
DSF137	*A. tennesseensis* NRRL 13150 (NR_135447.1)	98.49	6.90 ± 0.00 ^j^	14.93 ± 1.99 ^gh^	0.00 ± 0.00 ^l^	1.67 ± 2.89 ^f^	59.91 ± 7.62 ^d^
DSF138	*A. tennesseensis* NRRL 13150 (NR_135447.1)	99.25	0.00 ± 0.00 ^l^	0.00 ± 0.00 ^k^	0.00 ± 0.00 ^l^	15.39 ± 17.77 ^cdef^	41.46 ± 6.24 ^ef^
DSF148	*A. jensenii* NRRL 58600 (NR_135444.1)	97.77	0.00 ± 0.00 ^l^	0.00 ± 0.00 ^k^	0.00 ± 0.00 ^l^	0.00 ± 0.00 ^f^	34.78 ± 0.00 ^f^
DSF149	*T. resinae* (NR_190238.1)	96.04	56.38 ± 1.18 ^d^	16.01 ± 0.19 ^fgh^	44.04 ± 0.47 ^g^	32.93 ± 0.90 ^c^	76.85 ± 2.55 ^c^
DSF160	*A. sydowii* CBS 593.65 (NR_131259.1)	99.01	1.00 ± 1.73 ^kl^	0.00 ± 0.00 ^k^	0.00 ± 0.00 ^l^	0.00 ± 0.00 ^f^	47.22 ± 1.84 ^e^

Note: Values are expressed as mean ± standard deviation. Different lowercase letters within each column indicate significant differences at the 0.05 level. In the closest-match column, *A.*, *P.*, and *T.* represent *Aspergillus*, *Penicillium*, and *Talaromyces*, respectively. CH008, 171-1, and RC178 are strains of *C. gloeosporioides*; Pn006 represents *P. noxius*; and GP030 represents *G. pseudoferreum*. Closest-match names are provided for reference only and should not be interpreted as formal species-level identifications.

**Table 4:** Antifungal activity of sterile culture filtrates from nine selected fungal isolates against five phytopathogenic fungal strains.

Isolates	MGI (%)				
	**CH008**	**Pn006**	**171-1**	**RC178**	**GP030**
DSF005	38.62 ± 1.45 ^a^	0.00 ± 0.00 ^e^	−4.17 ± 3.61 ^d^	41.72 ± 3.24 ^a^	28.02 ± 6.05 ^c^
DSF007	9.19 ± 6.13 ^b^	16.46 ± 1.02 ^d^	0.00 ± 0.00 ^cd^	19.68 ± 4.87 ^bcd^	17.34 ± 3.45 ^c^
DSF012	39.42 ± 4.40 ^a^	50.38 ± 3.36 ^c^	40.31 ± 6.88 ^a^	26.15 ± 8.56 ^bc^	70.93 ± 6.47 ^a^
DSF044	33.55 ± 1.51 ^a^	84.09 ± 4.62 ^a^	11.67 ± 0.72 ^bc^	0.00 ± 0.00 ^e^	−15.72 ± 3.40 ^e^
DSF059	9.79 ± 4.62 ^b^	3.93 ± 4.14 ^de^	0.00 ± 0.00 ^cd^	8.48 ± 1.55 ^de^	−23.87 ± 7.08 ^ef^
DSF073	1.68 ± 2.90 ^b^	0.00 ± 0.00 ^e^	0.83 ± 1.44 ^cd^	0.00 ± 0.00 ^e^	25.98 ± 7.06 ^c^
DSF078	0.00 ± 0.00 ^b^	3.14 ± 2.96 ^e^	17.56 ± 7.32 ^b^	0.00 ± 0.00 ^e^	0.69 ± 1.20 ^d^
DSF109	30.89 ± 4.38 ^a^	66.56 ± 10.60 ^b^	6.87 ± 5.95 ^bcd^	30.08 ± 9.07 ^ab^	−35.21 ± 2.59 ^f^
DSF149	39.17 ± 4.77 ^a^	67.69 ± 1.13 ^b^	7.46 ± 4.97 ^bcd^	13.53 ± 2.93 ^cd^	44.99 ± 5.03 ^b^

Note: Values are expressed as mean ± standard deviation. Different lowercase letters within each column indicate significant differences at the 0.05 level. CH008, 171-1, and RC178 are three strains of *Colletotrichum gloeosporioides*; Pn006 represents *Phellinus noxius*; and GP030 represents *Ganoderma pseudoferreum*. Negative MGI values indicate that pathogen colony growth was greater than that of the corresponding control under the assay conditions.

**Table 5:** Effects of volatile emissions from nine selected fungal isolates on the mycelial growth of five phytopathogenic fungal strains in sealed-plate assays.

Isolates	MGI (%)				
	CH008	Pn006	171-1	RC178	GP030
DSF005	34.96 ± 0.00 ^a^	4.09 ± 0.83 ^ab^	51.17 ± 10.43 ^a^	−25.74 ± 0.00 ^c^	39.19 ± 6.20 ^bcd^
DSF007	42.35 ± 2.29 ^a^	0.00 ± 0.00 ^b^	0.00 ± 0.00 ^c^	−25.74 ± 0.00 ^c^	63.25 ± 7.13 ^a^
DSF012	10.72 ± 6.17 ^b^	6.79 ± 7.08 ^ab^	28.15 ± 24.91 ^abc^	10.37 ± 14.04 ^b^	28.58 ± 7.74 ^cd^
DSF044	50.69 ± 6.99 ^a^	0.00 ± 0.00 ^b^	0.81 ± 1.41 ^c^	−25.74 ± 0.00 ^c^	57.79 ± 10.26 ^ab^
DSF059	42.78 ± 12.51 ^a^	10.73 ± 2.46 ^b^	33.76 ± 8.99 ^ab^	4.96 ± 12.34 ^b^	47.18 ± 3.67 ^abc^
DSF073	50.80 ± 1.36 ^a^	0.00 ± 0.00 ^b^	14.68 ± 12.72 ^bc^	−25.74 ± 0.00 ^c^	62.84 ± 7.08 ^ab^
DSF078	32.44 ± 14.42 ^ab^	4.23 ± 3.69 ^ab^	36.03 ± 8.45 ^ab^	−3.23 ± 1.94 ^b^	0.00 ± 0.00 ^e^
DSF109	46.28 ± 8.93 ^a^	29.36 ± 5.08 ^a^	46.16 ± 0.89 ^a^	16.03 ± 6.86 ^ab^	0.83 ± 1.44 ^e^
DSF149	35.04 ± 9.63 ^a^	0.00 ± 0.00 ^b^	11.51 ± 4.32 ^bc^	36.28 ± 8.80 ^a^	19.06 ± 17.17 ^de^

Note: Values are expressed as mean ± standard deviation. Different lowercase letters within each column indicate significant differences at the 0.05 level. CH008, 171-1, and RC178 are three strains of *Colletotrichum gloeosporioides*; Pn006 represents *Phellinus noxius*; and GP030 represents *Ganoderma pseudoferreum*. Negative MGI values indicate that pathogen colony growth was greater than that of the corresponding control under the sealed-plate assay conditions.

## Data Availability

The ITS sequences generated in this study have been deposited in the GenBank database under accession numbers PQ302328–PQ302480 and PX857774–PX857779. Two priority isolates, DSF059 and DSF149, have been deposited in the China Center for Type Culture Collection (CCTCC, Wuhan, China) under accession numbers CCTCC No. M 20232310 and CCTCC No. M 20232230, respectively. Other purified isolates are maintained as cryopreserved stocks in the authors’ institutional culture collection and may be made available from the corresponding author upon reasonable request, subject to institutional and material-transfer regulations.

## Supplementary Materials

The following supporting information can be downloaded at: https://www.mdpi.com/article/10.3390/jof12080544/s1, Table S1: Sampling information for the deep-sea sediment materials used for fungal isolation. Table S2: Summary of the isolation protocols used for recovering culturable fungi from deep-sea sediment materials. Table S3: Composition of the culture media used in this study. Table S4: Identification and BLAST results of fungal isolates from deep-sea sediments, including closest GenBank matches, ITS similarities, accession numbers, and low-similarity phylotype labels. Table S5: Genus/phylotype-level composition and distribution of culturable fungal isolates recovered from deep-sea sediment materials based on ITS closest-match analysis. Figure S1: Full maximum-likelihood phylogeny inferred from ITS sequences of all 159 culturable fungal isolates recovered from deep-sea sediments and selected GenBank reference sequences. This ITS-based phylogeny should be interpreted as a preliminary placement framework rather than as robust species-level phylogenetic resolution. Figure S2: Principal coordinate analysis (PCoA) based on Bray–Curtis distances at the 57-taxon resolution: (A) sediment materials and (B) isolation protocols. Figure S3: Colony phenotypes of five phytopathogenic fungal strains grown on PDA amended with sterile culture filtrates from nine selected fungal isolates. Pathogen-only plates amended with sterile water served as controls. CH008, 171-1, and RC178 are three strains of *Colletotrichum gloeosporioides*; Pn006 represents *Phellinus noxius*; and GP030 represents *Ganoderma pseudoferreum*. Figure S4: Colony phenotypes of five phytopathogenic fungal strains exposed to volatile emissions from nine selected fungal isolates in base-to-base sealed-plate assays. Pathogen plates paired with uninoculated PDA plates served as controls. CH008, 171-1, and RC178 are three strains of *Colletotrichum gloeosporioides*; Pn006 represents *Phellinus noxius*; and GP030 represents *Ganoderma pseudoferreum*. Figure S5: Dual-culture colony phenotypes of five phytopathogenic fungal strains confronted with the remaining 14 deep-sea sediment-derived fungal isolates tested in the primary dual-culture antagonism assay. Pathogen-only plates served as controls. CH008, 171-1, and RC178 are strains of *Colletotrichum gloeosporioides*; Pn006 represents *Phellinus noxius*; and GP030 represents *Ganoderma pseudoferreum*.

## Abbreviations

The following abbreviations are used in this manuscript:

ANOVA	Analysis of variance
BIC	Bayesian information criterion
BLASTn	Basic Local Alignment Search Tool for nucleotide sequences
CTAB	Cetyltrimethylammonium bromide
EDTA	Ethylenediaminetetraacetic acid
GPY	Glucose-peptone-yeast extract agar
HSD	Honestly significant difference
ITS	Internal transcribed spacer
LSP	Low-similarity phylotype
MEA	Malt extract agar
MGI	Mycelial growth inhibition
PCoA	Principal coordinate analysis
PDA	Potato dextrose agar
SH-aLRT	Shimodaira–Hasegawa-like approximate likelihood ratio test
VOCs	Volatile organic compounds
