# Supplementary material for: Culturable Fungi Recovered from Deep-Sea Sediments: Diversity, Phylogenetic Placement, and In Vitro Antagonistic Activity Against Phytopathogenic Fungi
Source: J Fungi (Basel). 2026 Jul 23;12(8):544. doi: 10.3390/jof12080544 (PMC13514472; doi:10.3390/jof12080544)
Supplement: Supplementary file 1 [file jof-12-00544-s001.zip › Figure S1.pdf]

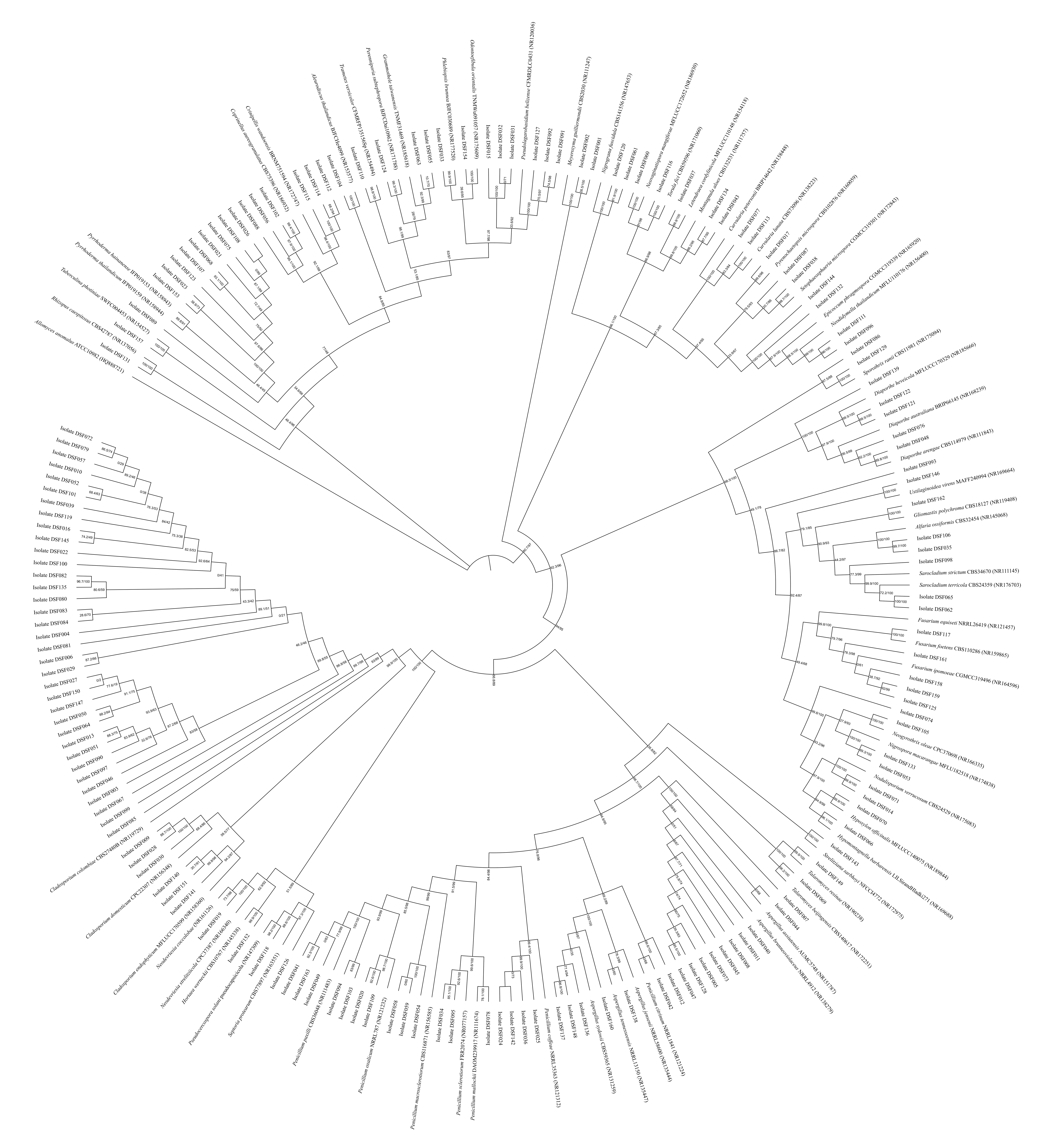
Isolate DSF072
Isolate DSF079
Isolate DSF057
Isolate DSF010
Isolate DSF052
Isolate DSF101
Isolate DSF039
Isolate DSF119
Isolate DSF016
Isolate DSF145
Isolate DSF022
Isolate DSF100
Isolate DSF082
Isolate DSF135
Isolate DSF080
Isolate DSF083
Isolate DSF084
Isolate DSF004
Isolate DSF081
Isolate DSF006
Isolate DSF029
Isolate DSF027
Isolate DSF150
Isolate DSF147
Isolate DSF050
Isolate DSF064
Isolate DSF013
Isolate DSF051
Isolate DSF090
Isolate DSF097
Isolate DSF046
Isolate DSF003
Isolate DSF067
Isolate DSF099
Isolate DSF085
Isolate DSF009
Isolate DSF028
Isolate DSF030
Cladosporium colombiae CBS27480B (NR119729)
Cladosporium domesticum CPC22307 (NR156348)
Isolate DSF140
Isolate DSF151
Isolate DSF141
Cladosporium endophyticum MFLUCC170599 (NR158360)
Neodevriesia coccolobae (NR161126)
Isolate DSF019
Neodevriesia strelitziicola CPC37387 (NR166340)
Hortaea werneckii CBS10767 (NR145338)
Isolate DSF152
Pseudocercospora solani pseudocapsicicola (NR147309)
Isolate DSF118
Septoria protearum CBS77897 (NR165551)
Isolate DSF126
Isolate DSF041
Isolate DSF163
Isolate DSF049
Penicillium paxilli CBS36048 (NR111483)
Isolate DSF094
Isolate DSF103
Isolate DSF020
Isolate DSF109
Penicillium oxalicum NRRL787 (NR121232)
Isolate DSF058
Isolate DSF059
Isolate DSF054
Penicillium macrosclerotiorum CBS116871 (NR156585)
Isolate DSF034
Isolate DSF095
Penicillium sclerotiorum FRR2074 (NR077157)
Penicillium mallochii DAOM239917 (NR111674)
Isolate DSF078
Isolate DSF024
Isolate DSF142
Isolate DSF036
Isolate DSF025
Penicillium coffeae NRRL35363 (NR121312)
Isolate DSF137
Isolate DSF148
Isolate DSF136
Aspergillus sydowii CBS59365 (NR131259)
Isolate DSF160
Aspergillus tennesseensis NRRL13150 (NR135447)
Isolate DSF138
Aspergillus jensenii NRRL58600 (NR135444)
Penicillium citrinum NRRL1841 (NR121224)
Isolate DSF042
Isolate DSF012
Isolate DSF047
Isolate DSF128
Isolate DSF005
Isolate DSF045
Isolate DSF073
Isolate DSF008
Isolate DSF011
Isolate DSF040
Aspergillus brunneoviolaceus NRRL4912 (NR138279)
Aspergillus assiutensis AUMC5748 (NR151787)
Isolate DSF044
Talaromyces beijingensis CBS140617 (NR172251)
Isolate DSF007
Isolate DSF069
Talaromyces resinae (NR190238)
Isolate DSF149
Strelitziana sarbhoyi NFCCI4772 (NR172975)
Isolate DSF143
Hypomontagnella barbarensis LJLStrandHladki271 (NR169688)
Isolate DSF066
Hypoxylon officinalis MFLUCC140075 (NR189844)
Isolate DSF070
Isolate DSF014
Isolate DSF071
Nodulisporium verrucosum CBS24529 (NR175083)
Isolate DSF053
Isolate DSF133
Nigrospora macarangae MFLU182518 (NR174838)
Neogyrothrix oleae CPC37069 (NR166335)
Isolate DSF105
Isolate DSF074
Isolate DSF125
Isolate DSF159
Isolate DSF158
Fusarium ipomoeae CGMCC319496 (NR164596)
Isolate DSF161
Fusarium foetens CBS110286 (NR159865)
Isolate DSF117
Fusarium equiseti NRRL26419 (NR121457)
Isolate DSF062
Isolate DSF065
Sarocladium terricola CBS24359 (NR176703)
Sarocladium strictum CBS34670 (NR111145)
Isolate DSF098
Isolate DSF035
Isolate DSF106
Alfaria ossiformis CBS32454 (NR145068)
Gliomastix polychroma CBS18127 (NR119408)
Isolate DSF162
Ustilaginoidea virens MAFF240994 (NR169664)
Isolate DSF146
Isolate DSF093
Diaporthe arengae CBS114979 (NR111843)
Isolate DSF048
Isolate DSF076
Diaporthe australiana BRIP66145 (NR168239)
Isolate DSF121
Isolate DSF122
Diaporthe heveicola MFLUCC170329 (NR185666)
Isolate DSF139
Sporothrix ranii CBS11981 (NR175094)
Isolate DSF129
Isolate DSF086
Isolate DSF096
Isolate DSF111
Neodidymella thailandicum MFLU110176 (NR156400)
Epicoccum phragmospora CGMCC319339 (NR165920)
Isolate DSF132
Isolate DSF144
Setophaeosphaeria microspora CGMCC319301 (NR172843)
Isolate DSF038
Isolate DSF087
Pyrenochaetopsis microspora CBS102876 (NR160059)
Isolate DSF017
Curvularia lunata CBS73096 (NR138223)
Isolate DSF113
Isolate DSF077
Curvularia petersonii BRIP14642 (NR158448)
Isolate DSF043
Isolate DSF134
Montagnula aloes CBS132531 (NR111757)
Letendraea cordylinicola MFLUCC10148 (NR154118)
Isolate DSF037
Torula fici CBS59596 (NR171060)
Isolate DSF116
Neovaginatispora mangiferae MFLUCC172652 (NR186930)
Isolate DSF060
Isolate DSF061
Isolate DSF120
Nigrograna fuscidula CBS141556 (NR147653)
Isolate DSF001
Isolate DSF002
Meyerozyma guilliermondii CBS2030 (NR111247)
Isolate DSF091
Isolate DSF092
Isolate DSF127
Pseudolagarobasidium belizense CFMRDLC0431 (NR120036)
Isolate DSF031
Isolate DSF032
Isolate DSF015
Odontoefibula orientalis TNMMW091057 (NR175609)
Isolate DSF154
Phlebiopsis brunnea BJFC030689 (NR175520)
Isolate DSF033
Isolate DSF055
Isolate DSF063
Grammothele taiwanensis TNMF31469 (NR185618)
Perenniporia substephospora BJFCDai10962 (NR171788)
Isolate DSF124
Trametes versicolor CFMRFP135156Sp (NR154494)
Isolate DSF110
Aleurodiscus thailandicus BJFCHe4009 (NR153577)
Isolate DSF104
Isolate DSF112
Isolate DSF114
Isolate DSF115
Crinipellis woodiorensis BRNM751594 (NR172747)
Coprinellus aureogranulatus CBS75396 (NR186932)
Isolate DSF102
Isolate DSF088
Isolate DSF056
Isolate DSF026
Isolate DSF108
Isolate DSF075
Isolate DSF021
Isolate DSF068
Isolate DSF107
Isolate DSF123
Isolate DSF023
Isolate DSF153
Pyrrhoderma hainanense IFP019153 (NR158943)
Pyrrhoderma thailandicum IFP019159 (NR158944)
Isolate DSF089
Tuberculina photiniae SWFC004453 (NR154527)
Isolate DSF157
Rhizopus caespitosus CBS42787 (NR137056)
Isolate DSF131
Allomyces anomalus ATCC10982 (HQ888721)
