# Supplementary material for: Culturable Fungi Recovered from Deep-Sea Sediments: Diversity, Phylogenetic Placement, and In Vitro Antagonistic Activity Against Phytopathogenic Fungi
Source: J Fungi (Basel). 2026 Jul 23;12(8):544. doi: 10.3390/jof12080544 (PMC13514472; doi:10.3390/jof12080544)
Supplement: Supplementary file 1 [file jof-12-00544-s001.zip › Figure S2.pdf]

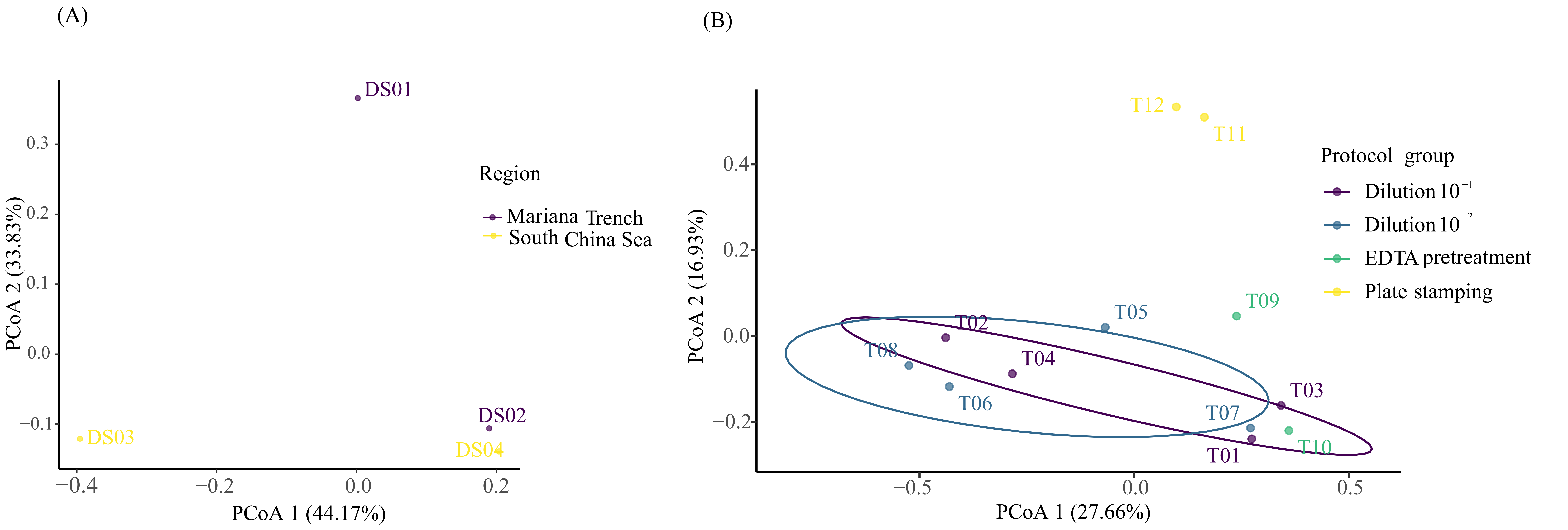

(A)
DS01
DS02
DS03
DS04
Region
Mariana Trench
South China Sea
PCoA 1 (44.17%)
PCoA 2 (33.83%)
(B)
T01
T02
T03
T04
T05
T06
T07
T08
T09
T10
T11
T12
Protocol group
Dilution 10⁻¹
Dilution 10⁻²
EDTA pretreatment
Plate stamping
PCoA 1 (27.66%)
PCoA 2 (16.93%)
