# Supplementary material for: Culturable Fungi Recovered from Deep-Sea Sediments: Diversity, Phylogenetic Placement, and In Vitro Antagonistic Activity Against Phytopathogenic Fungi
Source: J Fungi (Basel). 2026 Jul 23;12(8):544. doi: 10.3390/jof12080544 (PMC13514472; doi:10.3390/jof12080544)
Supplement: Supplementary file 1 [file jof-12-00544-s001.zip › Table S1.pdf]

Table S1. Sampling information for the deep-sea sediment materials used for fungal isolation.

Sample ID	Sampling region	Water depth (m)	Latitude (°N)	Longitude (°E)	Sediment fraction used for fungal isolation
DS01	Challenger Deep slope region, Mariana Trench	~6000	10.9523	141.9358	Bulk sediment material
DS02	Challenger Deep slope region, Mariana Trench	~5400	11.7640	141.9760	Columnar sediment material
DS03	Deep basin, South China Sea	2595	17.9167	112.5000	Mixed surface and deeper sediment material
DS04	Deep basin, South China Sea	2595	17.9167	112.5000	Middle sediment fraction
