# Supplementary material for: Culturable Fungi Recovered from Deep-Sea Sediments: Diversity, Phylogenetic Placement, and In Vitro Antagonistic Activity Against Phytopathogenic Fungi
Source: J Fungi (Basel). 2026 Jul 23;12(8):544. doi: 10.3390/jof12080544 (PMC13514472; doi:10.3390/jof12080544)
Supplement: Supplementary file 1 [file jof-12-00544-s001.zip › Table S2.pdf]

Table S2. Summary of the isolation protocols used for recovering culturable fungi from deep-sea sediment materials.

Protocol	Inoculation method	Pretreatment	Dilution	Medium
T01	Spread plate	—	10^{-1}	GPY
T02	Spread plate	—	10^{-1}	MEA
T03	Spread plate	—	10^{-1}	Fungus No. II
T04	Spread plate	—	10^{-1}	Martin
T05	Spread plate	—	10^{-2}	GPY
T06	Spread plate	—	10^{-2}	MEA
T07	Spread plate	—	10^{-2}	Fungus No. II
T08	Spread plate	—	10^{-2}	Martin
T09	Spread plate	EDTA pretreatment	10^{-1}	MEA
T10	Spread plate	EDTA pretreatment	10^{-2}	MEA
T11	Plate stamping	—	—	Fungus No. II
T12	Plate stamping	—	—	GPY
