# Supplementary material for: Culturable Fungi Recovered from Deep-Sea Sediments: Diversity, Phylogenetic Placement, and In Vitro Antagonistic Activity Against Phytopathogenic Fungi
Source: J Fungi (Basel). 2026 Jul 23;12(8):544. doi: 10.3390/jof12080544 (PMC13514472; doi:10.3390/jof12080544)
Supplement: Supplementary file 1 [file jof-12-00544-s001.zip › Table S3.pdf]

Table S3. Composition of the culture media used in this study (per 1 L of final medium).

Component	GPY	MEA	Fungus No. II	Martin	PDA
Glucose	20 g	–	10 g	10 g	20 g
Mannitol	–	–	20 g	–	–
Maltose	–	–	20 g	–	–
Malt extract powder	–	17 g	–	–	–
Peptone	10 g	3 g	–	5 g	–
Yeast extract	10 g	–	3 g	–	–
Sodium glutamate (MSG)	–	–	10 g	–	–
Corn steep liquor	–	–	1 g	–	–
Potato infusion (peeled)	–	–	–	–	200 g
K_2HPO_4	–	–	0.5 g	–	–
KH_2PO_4	–	–	–	1 g	–
$MgSO_4$	–	–	0.3 g	0.5 g	–
Rose bengal	–	–	–	0.03 g	–
Aged seawater	400 mL	400 mL	400 mL	400 mL	400 mL
Distilled water	to 1 L	to 1 L	to 1 L	to 1 L	to 1 L
Agar	20 g	20 g	20 g	17 g	15 g
Final pH	7.5	not adjusted	6.5	not adjusted	not adjusted
