# Supplementary material for: Culturable Fungi Recovered from Deep-Sea Sediments: Diversity, Phylogenetic Placement, and In Vitro Antagonistic Activity Against Phytopathogenic Fungi
Source: J Fungi (Basel). 2026 Jul 23;12(8):544. doi: 10.3390/jof12080544 (PMC13514472; doi:10.3390/jof12080544)
Supplement: Supplementary file 1 [file jof-12-00544-s001.zip › Table S5.pdf]

Table S5. Genus/phylotype-level composition and distribution of culturable fungal isolates recovered from deep-sea sediment materials based on ITS closest-match analysis.

Phylum	Class	Order	Genus / group	Number of isolates	Relative abundance (%)
Ascomycota	Sordariomycetes	Hypocreales	*Alfaria*	2	1.26
Ascomycota	Eurotiomycetes	Eurotiales	*Aspergillus*	15	9.43
Ascomycota	Dothideomycetes	Botryosporiales	*Botryosporium*	1	0.63
Ascomycota	Dothideomycetes	Cladosporiales	*Cladosporium*	41	25.79
Basidiomycota	Agaricomycetes	Agaricales	*Coprinellus*	1	0.63
Ascomycota	Dothideomycetes	Pleosporales	*Curvularia*	2	1.26
Ascomycota	Sordariomycetes	Diaporthales	*Diaporthe*	5	3.14
Ascomycota	Dothideomycetes	Pleosporales	*Epicoccum*	2	1.26
Basidiomycota	Exobasidiomycetes	Exobasidiales	*Exobasidiomycetidae*	1	0.63
Ascomycota	Sordariomycetes	Hypocreales	*Fusarium*	5	3.14
Ascomycota	Sordariomycetes	Hypocreales	*Gliomastix*	1	0.63
Ascomycota	Dothideomycetes	Capnodiales	*Hortaea*	1	0.63
Ascomycota	Sordariomycetes	Xylariales	*Hypoxylon*	1	0.63
Ascomycota	Dothideomycetes	Pleosporales	*Leptospora*	1	0.63
Ascomycota	Dothideomycetes	Pleosporales	*Letendraea*	1	0.63
Ascomycota	Saccharomycetes	Saccharomycetales	*Meyerozyma*	2	1.26
Ascomycota	Dothideomycetes	Pleosporales	*Montagnula*	1	0.63
Ascomycota	Dothideomycetes	Capnodiales	*Neodevriesia*	1	0.63
Ascomycota	Dothideomycetes	Pleosporales	*Neodidymella*	2	1.26
Ascomycota	Sordariomycetes	Xylariales	*Neogyrothrix*	1	0.63
Ascomycota	Dothideomycetes	Pleosporales	*Neovaginatispora*	1	0.63
Ascomycota	Dothideomycetes	Pleosporales	*Nigrograna*	2	1.26
Ascomycota	Sordariomycetes	Hypocreales	*Nigrospora*	2	1.26
Ascomycota	Sordariomycetes	Xylariales	*Nodulisporium*	2	1.26
Basidiomycota	Agaricomycetes	Polyporales	*Odontoefibula*	1	0.63
Ascomycota	Eurotiomycetes	Eurotiales	*Penicillium*	17	10.69
Basidiomycota	Agaricomycetes	Polyporales	*Perenniporia*	1	0.63
Ascomycota	Sordariomycetes	Xylariales	*Peroneutypa*	1	0.63
Basidiomycota	Agaricomycetes	Polyporales	*Phlebiopsis*	1	0.63
Ascomycota	Dothideomycetes	Capnodiales	*Pseudocercospora*	1	0.63
Ascomycota	Dothideomycetes	Pleosporales	*Pyrenochaetopsis*	1	0.63
Ascomycota	Dothideomycetes	Pleosporales	*Setophaeosphaeria*	1	0.63
Ascomycota	Eurotiomycetes	Eurotiales	*Talaromyces*	2	1.26
Ascomycota	Dothideomycetes	Pleosporales	*Torula*	1	0.63
Ascomycota	Sordariomycetes	Ustilaginoideales	*Ustilaginoidea*	1	0.63

—	—	—	Low-similarity phylotypes	37	23.27

Note: Relative abundance was calculated based on the total number of recovered isolates (*n = 159*).
